# Two-Component System Genes in *Sorghum bicolor*: Genome-Wide Identification and Expression Profiling in Response to Environmental Stresses

**DOI:** 10.3389/fgene.2021.794305

**Published:** 2021-11-25

**Authors:** Roshan Zameer, Muhammad Sadaqat, Kinza Fatima, Sajid Fiaz, Sumaira Rasul, Hadeqa Zafar, Abdul Qayyum, Naima Nashat, Ali Raza, Adnan Noor Shah, Riffat Batool, Farrukh Azeem, Sangmi Sun, Gyuhwa Chung

**Affiliations:** ^1^ Department of Bioinformatics and Biotechnology, Government College University, Faisalabad, Pakistan; ^2^ Department of Plant Breeding and Genetics, The University of Haripur, Haripur, Pakistan; ^3^ Institute of Molecular Biology and Bio-Technology, Bahauddin Zakariya University, Multan, Pakistan; ^4^ Nuclear Institute for Agriculture and Biology, Faisalabad, Pakistan; ^5^ Department of Agronomy, The University of Haripur, Haripur, Pakistan; ^6^ Department of Biochemistry, University of Agriculture, Faisalabad, Pakistan; ^7^ Fujian Provincial Key Laboratory of Crop Molecular and Cell Biology, Oil Crops Research Institute, Center of Legume Crop Genetics and Systems Biology, College of Agriculture, Fujian Agriculture and Forestry University (FAFU), Fuzhou, China; ^8^ Department of Agricultural Engineering, Khwaja Fareed University of Engineering and Information Technology, Rahim Yar Khan, Pakistan; ^9^ Department of Botany, GC Women University, Faisalabad, Pakistan; ^10^ Department of Biotechnology, Chonnam National University, Yeosu, South Korea

**Keywords:** signaling, TCS, HKS, Rrs, qRT-PCR

## Abstract

The two-component signal transduction system (TCS) acts in a variety of physiological processes in lower organisms and has emerged as a key signaling system in both prokaryotes and eukaryotes, including plants. TCS genes assist plants in processes such as stress resistance, cell division, nutrition signaling, leaf senescence, and chloroplast division. In plants, this system is composed of three types of proteins: response regulators (RRs), histidine kinases (HKs), and histidine phosphotransfer proteins (HPs). We aimed to study the *Sorghum bicolor* genome and identified 37 *SbTCS* genes consisting of 13 HKs, 5 HPs, and 19 RRs (3 type-A RRs, 7 type-B RRs, 2 type-C RRs, and 7 pseudo-RRs). The structural and phylogenetic comparison of the *SbTCS* members with their counterparts in *Arabidopsis thaliana*, *Oryza sativa*, *Cicer arietinum*, and *Glycine max* showed group-specific conservations and variations. Expansion of the gene family members is mostly a result of gene duplication, of both the tandem and segmental types. HKs and RRs were observed to be originated from segmental duplication, while some HPs originated from tandem duplication. The nuclear genome of *S. bicolor* contain 10 chromosomes and these *SbTCS* genes are randomly distributed on all the chromosomes. The promoter sequences of the *SbTCS* genes contain several abiotic stress-related *cis*-elements. RNA-seq and qRT-PCR-based expression analysis demonstrated most of the *TCS* genes were responsive to drought and salt stresses in leaves, which suggest their role in leaf development. This study lays a foundation for further functional study of *TCS* genes for stress tolerance and developmental improvement in *S. bicolor.*

## 1 Introduction

The two-component system (TCS) was first recognized in bacteria, and it has since been studied for signal transduction pathways in fungi, slime molds, and plants (Stock et al., 2000). As the name suggests, this system involves two primary components in bacteria: a sensor histidine kinase (HK) and a response regulator (RR) (Capra and Laub 2012). The sensor HK contains two domains: the input domain and the transmitter domain. The input domain perceives signals and, as a result, the HK activity of the transmitter domain is modified after autophosphorylation (kinase self-activation) of a conserved histidine (H) residue. In the receiver domain of the RR (Rec), the phosphate group is then transferred to a conserved Asp residue (Aspartic acid). Another domain, known as the output domain, is also found in many RRs; its activity is regulated by the phosphorylation state of the Rec domain whereas, Rec domain is a response regulator receiver domain. The output domain commonly behaves as a transcription factor (Sun et al., 2014). Over time, an additional protein family, whose members are histidine phosphotransfer proteins (HPs), has been found to be involved in developing a multi-step phosphorylation mechanism in eukaryotes. In the corresponding signaling cascade, a phosphate group is transferred among the HP family members, and may involve as a linker between HKs and RRs proteins (Wurgler-murphy 1997; West et al., 2001). In plants such as *Arabidopsis thaliana*, there are three distinct subfamilies of HKs: ethylene receptor, phytochrome receptor, and cytokinin receptors (Hwang et al., 2002). Three additional *A. thaliana* HKs (hybrid HKs; AHK1, ACKl1, and CKl2/AHK5) belong to no known group. The overall structure of an HK involves an input domain, a Rec domain, several transmembrane domains (at the N-terminus), and a conserved H residue containing a transmitter domain (the autophosphorylation site). However, due to a lack of conserved residues and motifs, three ethylene receptors (AETR2, ERS2, and AEIN4) and phytochromes cannot perform HK activity; therefore, they are referred to as divergent HKs (Ahmad et al., 2020). The ethylene receptor family has an ethylene binding transmembrane domain at the N-terminal, a His protein kinase domain, and a GAF protein-protein interaction domain (Chen et al., 2020). ERS1 and ETR1 are two further subdivisions of the ethylene receptor family based on the similarity of their amino acid sequences. In *A. thaliana*, the ethylene receptor family consists of five members (ETR1, ETR2, EIN4, ERS1, and ERS2), which contain an ethylene (C_2_H_4_) binding domain (Dhar et al., 2019). Phytochromes/photoreceptors are involved in the regulation of plant growth and development in response to light stimuli (Paik and Huq 2019). PHYA, PHYB, PHYC, PHYD, and PHYE are the five phytochrome receptors present in *A. thaliana*; they contain two main structural domains: the amino-terminal domain and the carboxyl terminus. A linear tetrapyrrole chromophore is covalently attached to the amino-terminal domain for light absorption and photoreversibility. For signal transduction, the carboxyl terminus contains two PAS domains and a His protein kinase-related domain. Moreover, AHK2, AHK3, and AHK4 are considered cytokinin receptors, which are recognized on the basis of containing the cyclase/HK-associated sensing extracellular (CHASE) domain (Hutchison and Kieber, 2002).

The HP family contains a domain called the phosphotransfer (Hpt) domain which is essential for transferring a phosphate group from the Rec domain of HKs to the Rec domain of RRs, enabled due to the presence of a highly conserved motif (XHQXKGSSXS) (Gupta et al., 2020; He et al., 2020; Tiwari et al., 2021). The *AHP1*–*AHP5* are the five *A. thaliana* genes that encode the intermediate proteins with an Hpt domain. However, AHP6 lacks an H residue of that motif; as a consequence, it is called pseudo-His phosphotransfer protein (pseudo-Hp). Furthermore, AHP6 cannot behave as a phosphotransfer protein; thus, it is considered a cytokinin signaling negative regulator. Based on the domains, signal nature, and conserved sequences, the RR family is divided into three subgroups: type-A, type-B, and type-C RRs. Type-A RRs are cytokinin response proteins that have a Rec domain and a C-terminal extension. Type-B RRs consist of an N-terminal Rec and a C-terminal output domain. Type-C RRs have domain structures similar to those of type-A RRs, but are induced by cytokinin. Type-C RRs have still not been reported to have a role in cytokinin signaling. There is another distinct class of RRs, known as pseudo-RRs (PRRs), which lack a highly conserved phosphor-accepting aspartate (D) residue that is needed for phosphorylation. The CCT (Co, Col, and Toc1) motif in the C-terminal extension of PRRs is necessary for regulating circadian rhythms. Although PRRs are not involved in the transduction of phosphorelay signals, they play key roles in the circadian clock, which is implicated in a variety of distinct signal transduction processes (light-stimulated) in plants (Ishida et al., 2009; Tsai et al., 2012). The TCS is studied in various prokaryotes and eukaryotes, including plants for signal transduction pathways (Mizuno 2005; Tiwari et al., 2017). The *TCS* genes play a significant role in various abiotic stress responses, including to different temperature, water, and salinity conditions (He et al., 2016a). Signal transduction in plants is mediated by the TCS, which is also involved in osmosensing and essential cellular processes such as responses to ethylene, cytokinin, and red light. Studies on TCS in an *A. thaliana* model plant have been carried out and have led to unprecedented advances in our understanding of the circadian clock and the mechanisms of plant hormonal responses (i.e., ethylene and cytokinin responses). the TCS is involved in processes such as nutrient sensing, stress response, chemotaxis, endosperm formation, and nodulation during plant development, growth, and adaptation (Ishida et al., 2009; Zwack and Rashotte 2015). Several *A. thaliana* TCS genes work together with ABA to adapt to low temperature, drought, and salt stresses. *AHP1*, *AHP2*, and *AHP3* are highly expressed in heat stress conditions (Miyata et al., 1998). In *O. sativa*, drought and salt stresses affect *OsAHP1/2* knockdown seedlings in various ways. Similarly, ABA-induced antioxidant defense occurs *via OsHK3* (Sun et al., 2014). In *G. max*, dehydration affects the expression level of most *TCS* genes (Le et al., 2011). Some *TCS* genes of tomatoes are active in stress response. Pollen from the tomato *Never-ripe* (*Nr*) HK mutant is highly susceptible to heat stress. Some phytochromes act as HKs, helping plants respond to drought stress (Firon et al., 2012).

Sorghum is a C4 grass that ranks fifth in terms of acreage after wheat, *Z. mays*, *O. sativa*, and barley, with a world annual production of approximately 65.5 million tons derived from 45 million ha (Dicko et al., 2006). It is a self-pollinating and extensively grown cereal crop that adapts to various purposes, resulting in phenotypic variations between varieties. The genetic and phenotypic diversity of *S. bicolor* has increased due to its widespread distribution across Asia, India, the Middle East, and Africa, which has resulted in divergent botanical types mainly characterized by their seed characteristics and floral architecture. This crop is grown in both subsistence and commercial agriculture systems worldwide for fuel, fiber, food (syrup and grain), and animal feed. Since the rise in temperature and salinity are two major constraints having multidimensional impact on plant growth and development. So the crop improvement must be accelerated to meet the expected global food demand over the next few decades (Morris et al., 2013; Cooper et al., 2019). The objective of our current study was to identify the drought and salt stress responsive TCS genes that are potentially useful for *S. bicolor* breeding. Since the TCS genes are involved in several biological processes, it is essential to thoroughly investigate these genes in *S. bicolor.*

## 2 Materials and Methods

### 2.1 Identification of Two-Component System Gene Family in *Sorghum bicolor*

Firstly, the *A. thaliana* full-length protein sequences of TCS were retrieved from Ensemble plants database (http://plants.ensembl.org/index.html). These sequences were used as query sequence to execute a BLASTp program search to identify the TCS gene family members in *S. bicolor*. All putative sequences were further evaluated to check the presence of a specific domain using different domain databases including, CDD (https://www.ncbi.nlm.nih.gov/Structure/cdd/wrpsb.cgi) (Marchler-Bauer et al., 2011), SMART (http://smart.embl-heidelberg.de/) (Letunic et al., 2012) and Pfam (https://pfam.xfam.org/) (Punta et al., 2012). This step was taken to eliminate the sequences that lacked specific conserved domains required for TCS protein function. The sequences were manually sorted to remove the redundancy, and the remaining proteins were considered as identified TCS proteins. Molecular weight (MW), theoretical isoelectric point (pI), aliphatic index, instability index and the grand average of hydrophobicity (GRAVY) values were calculated by using the online tool ProtParam from ExPASY server (https://web.expasy.org/protparam/) (Gasteiger et al., 2003). In addition, the subcellular localization of SbTCS genes was determined by using online CELLO v.2.5 (http://cello.life.nctu.edu.tw/) (Yu et al., 2006).

### 2.2 Phylogenetic Analysis, Genetic Structure and Conserved Motif

To comprehend the SbTCS genes’ evolutionary relationship, multiple sequence alignment of the identified TCS proteins of *S. bicolor,* and already reported sequences of *A. thaliana, C. arietinum O. sativa, and G. max* was performed using ClustalW tool, and the neighbor-joining (NJ) tree was created using MEGA7 (https://www.megasoftware.net/) (Kumar et al., 2016) with a bootstrap value of 1,000. The exon and intron structures were visualized using the online software Gene Structure Display Server (GSDS) (http://gsds.gao-lab.org/) through matching the genomic sequences and coding sequences (CDS) of identified TCS genes, which were retrieved from NCBI (Hu B. et al., 2015). Furthermore, the MEME (Multiple EM for Motif Elicitation) tool (https://meme-suite.org/meme/tools/meme) was used to predict the specific conserved motifs of each TCS protein sequence (Bailey et al., 2015). The maximum number of motifs was set to 20, and other parameters were at the default setting.

#### 2.2.1 *Cis*-Regulatory Elements and Gene Ontology Analysis

The upstream 1,000 bp genomic DNA sequences from the transcription start site of *SbTCS* genes were extracted from NCBI. Then they were submitted to an online plantCARE database (http://bioinformatics.psb.ugent.be/webtools/plantcare/html/) to predict the putative *cis*-regulatory elements (Rombauts et al., 1999). Entrez gene id of TCS genes was used for gene ontology enrichment analysis using the open-access DAVID Bioinformatics Resources 6.7.

### 2.3 Chromosomal Localization, Gene Duplication Events and Syntenic Analysis

By using TBtool advance Circos, a genetic relation map of chromosomes was constructed. Moreover, NCBI-gene database was used to predict the position of each TCS gene on the chromosomes of *S. bicolor* (Yu et al., 2006; Brown et al., 2015). The syntenic analysis was conducted using TBtool (Chen et al., 2018). DnaSP v.6 tool was utilized to analyze the gene duplication events. Synonymous and non-synonymous substitution rates were calculated to determine the selective pressure on the duplicated genes (Rozas et al., 2017). Divergence time was also calculated to perceive the evolutionary events. The following formula calculated the duplication time: *T* = Ks/2*x* (*x* = 6.56 × 10^–9^) (He et al., 2016b).

### 2.4 Expression Patterns of Two-Component System Genes in *Sorghum bicolor*

To understand the expression pattern of these identified SbTCS*s* under saline/alkali and drought stress, RNA-seq data (BioProject: PRJNA319738 for drought stress tolerance and BioProject: PRJNA591555 for saline/alkali stress) was downloaded from NCBI Sequence Read Archive (SRA) database (https://www.ncbi.nlm.nih.gov/sra). The genome annotation in .fna and .gtf extension were downloaded from (https://www.ncbi.nlm.nih.gov/assembly/GCF_000003195.3/). Indexes of *S. bicolor* genome sequence were built using bowtie2, and paired-end clean reads with high quality were mapped to the *S. bicolor* genome. The expression level of the annotated genes in the reference genome was then calculated by the cufflinks program. The normalized FPKM (fragment per kilobase of transcript per million fragments mapped reads) values of each SbTCS were calculated, and differentially expressed genes were identified. The heatmap was generated to envision the expression through TBtool (Chen et al., 2018).

### 2.5 Plant Growth and Treatments

Plants (*S. bicolor,* JS2002) were grown for 28 days in a growth chamber under controlled conditions: 25–27°C day-night temperature with 12-h light and 65% humidity. Plants were exposed to drought stress (well-watered and limited water supply), 10 mM or moderate saline-alkali soil stress (6 and 24 h), and 50 mM or severe saline-alkali soil stress (6 and 24 h). The Saline-alkali solution was used to apply saline-alkali soil stress; this solution was made of Na_2_CO_3_ and NaHCO_3_ (1:9, v/v) with half-strength Hoagland’s nutrient solution including Na^+^ at 150 mM and pH 9.5 (Hu G. et al., 2015). 3% salt content were used for moderate and 5% salt content were used for severe condition. For RNA extraction purposes, leaf samples were collected from all the pots (Control, Drought, moderate saline-alkali, and severe saline-alkali) with three biological replicates and then speedily frozen in liquid nitrogen and kept at −80°C until further use.

### 2.6 Validation of Quantitative Real Time PCR

In the presence of liquid nitrogen, leaf samples were grounded into fine powder by using sterile pestles and mortar. By using the Fastlane cell cDNA kit the complementary DNA (cDNA) was synthesized (Qiagen, Switzerland). A Nanodrop spectrophotometer (NanoDrop 2000 spectrophotometer, Thermo Fisher Scientific) was used to quantify RNA. The qPCR reactions were executed in Applied Biosystem Real-Time PCR Detection System using SYBR Green Master kit (Applied Biosystems, United Kingdom). Gene-specific primers were designed through the online tool “Oligo Calculator” (http://mcb.berkeley.edu/labs/krantz/tools/oligocalc.html), and specificity of these primers was then confirmed by NCBI Primer-BLAST program (https://www.ncbi.nlm.nih.gov/tools/primer-blast/) (Supplemental Table S1). The glyceraldehyde-3-phosphate dehydrogenase (GAPDH) gene has been used as the reference gene to normalize gene expression (Sudhakar Reddy et al., 2016).

## 3 Results

### 3.1 Comprehensive Identification of Two-Component System Genes in *Sorghum bicolor*

A BLASTp search was performed to identify the putative members of the TCS gene family in *S. bicolor* by employing 47 *A. thaliana* TCS query protein sequences. A total of 37 TCS genes were identified in the genome of *S. bicolor*, which were further divided into 13 HKs, 5 HPs, and 19 RRs.

### 3.2 Histidine Kinase Protein Family in *Sorghum bicolor*

**FIGURE 1:**
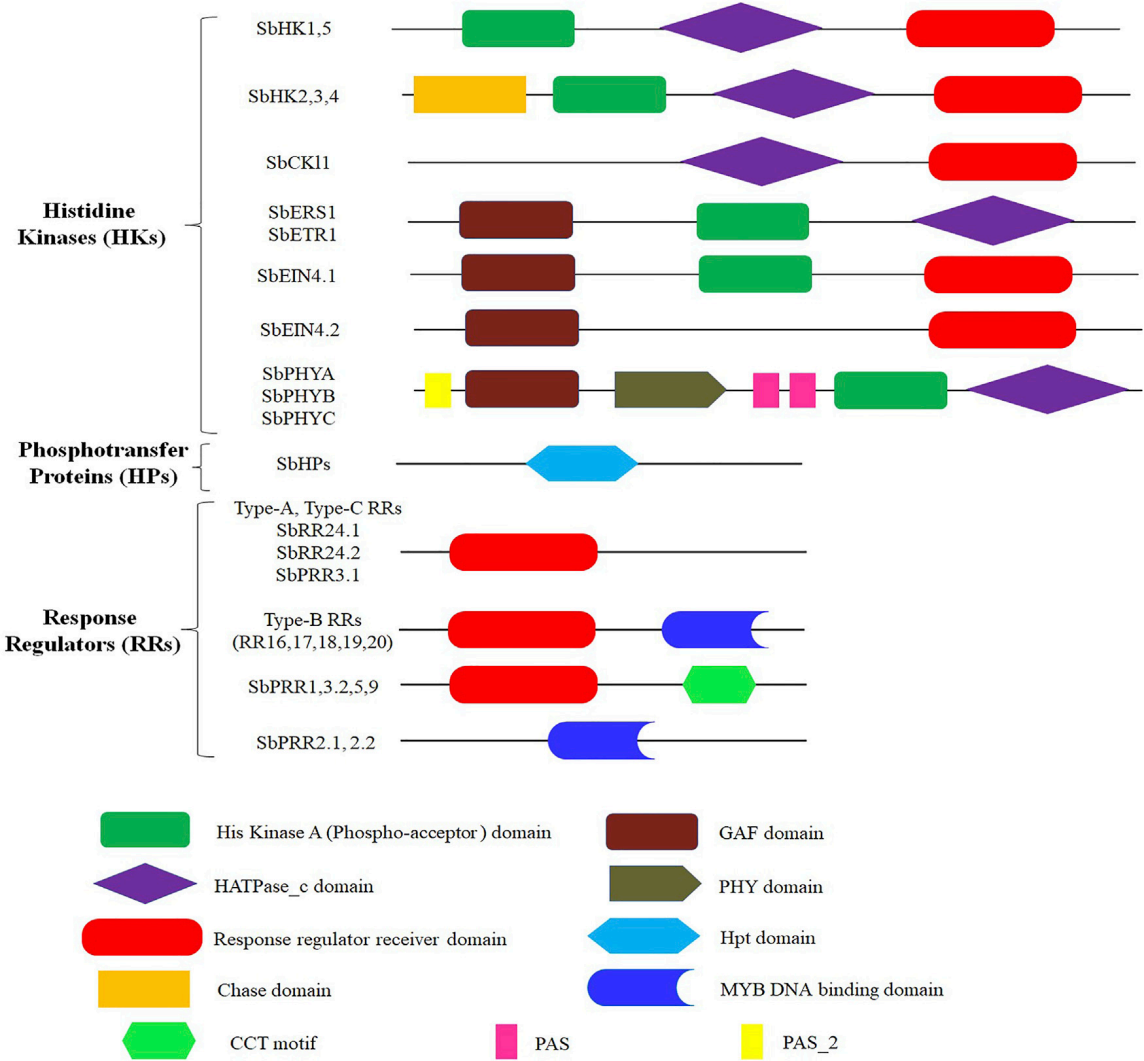
Symbolic domain structure of *S. bicolor* two component signaling elements, different shapes and color represents different domains.

The genome of *S. bicolor* contains 13 HKs (SbHKs/SbHKLs). This number is comparatively more significant than those present in *Oryza sativa L.* (5), *Zea mays* (11), *Triticum aestivum* (7), and *Populus trichocarpa* (12)*.* However, other legumes such as *A. thaliana* (17), *Lotus japonicas* (14), *Cucumis melo L.* (17), and *Physcomitrella patens* (18) have a similar number of HKs, which reveals their significance among plants (Table 1). We identified six genes encoding members of the cytokinin receptor family in *S. bicolor*, i.e., *SbHK1*, *SbHK2*, *SbHK3*, *SbHK4*, *SbHK5*, and *SbCKl1*. The conserved residues required for HK activity were present in all members. Domain analysis of these HKs confirmed that five members (except for *SbCKl1*) contained a conserved HisKa domain, having a conserved His phosphorylation site. Moreover, all six members had a conserved RR (Rec) domain in which a highly conserved Asp, which acts as the photoreceptor, is present (Figure 1; Supplementary Table S2, ). Genetic and molecular analyses of *A. thaliana* have shown that five ethylene receptors are involved in ethylene response. These receptors have a GAF protein-protein interactive domain, a HisKa domain, and a HATPase_c domain. Similarly, four genes encoding ethylene receptors were predicted in *S. bicolor* (*SbERS1*, *SbETR1*, *SbEIN4.1*, and *SbEIN4.2*). Of these, *SbERS1* and *SbETR1* encoded almost the same domains as those of *A.thaliana. SbEIN4.1* was found to contain GAF, HisKa, and a conserved Rec domain, whereas *SbEIN4.2* contained a GAF and a conserved Rec domain (Figure 1; Supplementary Table S2).

**TABLE 1:** Summary of the identified TCS gene members from different plants.

Species	Cotyledons	HK	HP (Pseudo-HP)	Type-A RR	Type-B RR	Type-C RR	Pseudo RR	Total	References
*Cucumis melo L.*	Eudicots	17	9	8	11	0	6	51	Liu et al. (2020)
*Arabidopsis thaliana*	Dicot	8	6 (1)	10	12	2	9	47	Hutchison and Kieber (2002)
*Citrullus lanatus*	Dicot	19	6 (2)	8	10	1	5	49	He et al. (2016b)
*Cucumis sativus L.*	Dicot	18	7 (2)	8	8	0	5	46	He et al. (2016b)
*Lotus japonicus*	Dicot	14	7	7	11	1	5^a^	40	Ishida et al. (2009)
*Glycine max*	Dicot	36	13	18	15	3	13	98	Mochida et al. (2010)
*Cicer arietinum*	Dicot	18	7 (2)	7	7	2	10^b^	51	Ahmad et al. (2020)
*Populus trichocarpa*	Dicot	12	12	9	11	0	5	49	Singh and Kumar (2012)
*Brassica rapa*	Dicot	20	8 (1)	21	17	4	15	85	Liu et al. (2014)
*Solanum lycopersicum*	Dicot	20	6 (2)	7	23	1	8	65	Firon et al. (2012)
*Triticum aestivum*	Monocot	7	10	41	2	0	2	62	Gahlaut et al. (2014)
*Physcomitrella patens*	Monocot	18	3	7	5	2	4^a^	39	Ishida et al. (2010)
*Zea mays*	Monocot	11	9 (2)	16	9	3	11^a^	59	Chu et al. (2011)
*Oryza sativa L.*	Monocot	5	5	15	7	0	5	37	Du et al. (2007)
*Zizania latifolia*	Monocot	25	8	14	14	2	6	69	He et al. (2020)
*Sorghum bicolor*	Monocot	13	5 (2)	3	7	2	7^b^	37	Present Work

a Only clock-associated.

b Both clock associated and type-B PRRs.

Members of the phytochrome family/photoreceptors allow plants to regulate their growth and developmental process and respond to light stimuli. *PHYA*, *PHYB*, *PHYC*, *PHYD*, and *PHYE* are the members of this family that have been reported in *A. thaliana*. Phytochromes contain a PHY domain at the N-terminal (involved in the absorption of light), a GAF domain, one HisKa, and two PAS domains involved in the transduction of signals. Sensor H proteins have a similar structure to that of phytochromes, which are soluble proteins. They have a signal transduction HisKa domain at the C-terminus and a sensor domain at the N-terminus. Since the phytochrome family lacks all five conserved motifs, they are referred to as divergent HKs. Instead of having HK activity, they have another Ser/Thr kinase activity (Ahmad et al., 2020). Three members of the phytochrome family were found in *S. bicolor* (*SbPHYA*, *SbPHYB*, and *SbPHYC*). All the domains (PAS, PHY, GAF, and HisKa domains) necessary for the regulation of light and signal transduction were present in their protein products, making them actual photoreceptors.

### 3.3 Histidine Phosphotransfer Proteins Family

Five out of six members of the *A. thaliana* HP (*AHP1*–*AHP6*) family are true HPs; one member, AHP6, is considered a pseudo-HP due to the absence of a conserved residue (H) required to obtain phosphate from the donor proteins (Bleecker 1999). Except for *AHP6*/*APHP1*, all the other members encode a conserved phosphorylation motif (XHQXKGSSXS). In AHP6/APHP1, the N residue has replaced the H residue of the phosphorylation motif. In *S. bicolor*, five members of the HP family were discovered to have a conserved domain, “Hpt” (Figure 1; Supplementary Table S2). Three of these five members (*SbHP1*, *SbHP2*, and *SbHP5*) were classified as true HPs since they have the phosphorylation motif, while the other two members (*SbHP3* and *SbHP4*) were considered pseudo-HPs because the H residue is missing from their phosphorylation motif.

### 3.4 The Response Regulators

RRs modulate the final responses to different environmental stimuli. In this study, 19 RR family members were identified in *S. bicolor*, including both standard and pseudo-RRs. Previous studies have reported that *A. thaliana* has 32 members of the RR family (Mochida et al., 2010) and that *O. sativa* has 22 (Du et al., 2007). RRs act as terminal components in the TCS signaling pathway, acting as phosphorylation-activated switches. This RR functioning catalyzes the transport of phosphoryl groups to the Asp residue of its conserved domain. A significant feature of RRs is that they contain conserved K (lysine) and D (aspartic acid) residues, which are found in the Rec domain of *A. thaliana* RR family members. On the basis of the domains that are conserved, RR family members are further classified into three subfamilies: type-A RRs, type-B RRs, and type-C RRs. Type-A RRs have a Rec domain with a conserved D residue and a C-terminal extension. The Rec and DNA binding domains (Myb) are present in type-B RRs. Type-C RRs have a similar structure to that of type-A RRs but do not have a C-terminal extension. Another type of RR is known as the pseudo-response regulator (PRR). These RRs have a conserved Rec domain with an E residue (in place of the D residue) and a C-terminal CCT motif (Ahmad et al., 2020). Among the 19 *S. bicolor* RR family members, 2, 3, 7, and 7 are type-C RRs, type-A RRs, PRRs, and type-B RRs, respectively.

Three members of the type-A RR family in *S. bicolor* were identified, namely *SbRR4*, *SbRR9*, and *SbRR10*. These putative members of the type-A RR family were similar to their *A. thaliana* counterparts, with all three possessing a conserved Rec domain. Nuclear proteins are the most common members of the type-B RR family. These are different from the members of the type-A RR family since they contain a Myb-DNA binding domain. It has been reported that type-B RRs act as transcription factors (Ahmad et al., 2020). Seven members of this family were identified in *S. bicolor* (*SbRR16*, *SbRR17*, *SbRR18*, *SbRR19, SbRR20*, *SbRR24.1*, and *SbRR24.2*). This number is higher than that in *T. aestivum* (2) and lower than that in many other plant members, including *A. thaliana* and *O. sativa.* All seven members were found to have conserved Rec and Myb domains, except for *SbRR24.1* and *SbRR24.2*, from which the Myb domain is missing. In *A. thaliana*, there are two members of the type-C RR family (*ARR22* and *ARR24*). Like type-A RRs, they also have a conserved Rec domain, but possess a very small C-terminal. They are not very closely related to type-A RRs, according to phylogenetic analysis. It has also been shown that they are not expressed during cytokinin response. *S. bicolor* was found to contain two members of the type-C RR family (*SbRR13* and *SbRR14*). These members exhibited significant homologous relationships with their *A. thaliana* counterparts and possessed a conserved Rec domain.

Divergent RRs, also known as PRRs, are another type of RRs found in a variety of plant species. In *A. thaliana*, there are nine divergent RR family members (*ARR1*–*9*). They have the entire Rec domain but lack a conserved DDK motif. Seven PRRs were found in the *S. bicolor* genome: *SbPRR1*, *SbPRR2.1*, *SbPRR2.2*, *SbPRR3.1*, *SbPRR3.2*, *SbPRR5*, and *SbPRR9*. PRRs have been further divided into two groups based on the C-terminal extension: type-B PRRs and clock-associated PRRs. In type-B PRRs (*SbPRR2.1* and *SbPRR2.2*), instead of the CCT motif, a Myb domain is present. While clock-associated PRRs (*SbPRR1*, *SbPRR3.2*, *SbPRR5*, and *SbPRR9*) have the conserved amino acids arginine and lysine in the CCT motif, in *SbPRR3.1*, only a pseudo-rec domain is present.

### 3.5 Features of the SbTCS Proteins

The detailed physio-chemical characteristics of 37 SbTCS proteins are shown in Table 2 and Supplementary Table S4. The SbTCSs are present in 10 chromosomes (Chr), Chr 1–10. The exon numbers ranged from 2 to 14. The protein length ranged from 132 (*SbRR13*) to 1,178 (*SbPHYB*) amino acids (aa). ExPASy analysis revealed that the SbTCS proteins exhibited very different isoelectric point (pI) values (ranging from 4.55 to 9.69), molecular weight values (ranging from 14,169.76 to 122,065.35), aliphatic index values (ranging from 57.62 to 106.71), and grand average of hydropathicity index (GRAVY) values (ranging from −0.827 to 0.224). SbTCS proteins were shown to be located in the cytoplasmic, mitochondrial, and nuclear membranes.

**TABLE 2:** Features of cytokinin two component signaling system genes in *S. bicolor.*

Gene name	Gene symbol	Chr	Start site	End site	CDS	Exon	Introns	Protein length	Molecular weight(MW)	Isoelectric point(PI)	Instability index(II)	Aliphatic index	Grand average of hydropathicity (GRAVY)	Cell location
Histidine kinases (HKs)														
SbHK1	LOC8059279	1	22266130	22269754	2451	5	4	743	8,0885.46	5.96	48.81-unst	91.33	−0.076	Cytoplasmic
SbHK2	LOC8075742	4	59266327	59273161	4149	10	9	973	108,350	5.91	38.85	87.58	−0.179	Plasma membrane
SbHK3	LOC110433965	3	71489995	71495445	3709	11	10	1,005	112,373.2	8.42	38.35	90.22	−0.226	Extracellular
SbHK4	LOC8060004	1	8870876	8878374	3669	11	10	996	109,153.9	5.51	46.05-unst	91.66	−0.09	Cytoplasmic
SbHK5	LOC8069360	10	55655919	55666166	4985	14	13	959	108,270.6	5.34	50.78-unst	80	−0.508	Nuclear
SbCKl1	LOC8076204	10	5136492	5152208	3457	8	7	1,138	122,065.4	5.63	42.59-unst	91.31	−0.055	Plasma membrane, cytoplasmic, mitochondrial
SbERS1	LOC8063415	1	9801882	9806982	2470	6	5	635	70,635.29	7.07	37.87	106.71	0.144	Plasma membrane
SbETR1	LOC8076011	9	5018113	5022906	2920	6	5	632	70,153.82	6.84	37.48	106.19	0.144	Plasma membrane
SbEIN4.1	LOC8155512	4	67810845	67814687	3633	3	2	899	99,732.68	8.28	unst-50.1	100.46	0.078	Plasma membrane
SbEIN4.2	LOC8066019	6	3178416	3182964	3346	3	2	773	85,953.83	7.04	36.79	102.41	0.088	Plasma membrane
SbPHYA	LOC8059991	1	8713754	8721151	3941	6	5	1,131	125,094.3	5.77	unst-49.69	92.6	−0.151	Cytoplasmic
SbPHYB	LOC8081072	1	68035215	68043712	4750	4	3	1,178	129,046.6	5.75	unst-48.61	86.72	−0.167	Cytoplasmic, plasma membrane
SbPHYC	LOC8086232	1	6748035	6753340	4292	4	3	1,135	126,205	5.72	unst-51.31	97.24	−0.112	Cytoplasmic
Phosphotransfer Proteins (HPs)														
SbHP1	LOC8062536	2	19797592	19801117	1,132	6	5	145	16,228.54	4.55	40	91.52	−0.079	Nuclear, extracellular, cytoplasmic
SbHP2	LOC8057993	7	60641495	60644235	824	6	5	144	16,172.66	5.63	26.55	88.68	−0.089	Nuclear, extracellular
SbHP3	LOC8075559	3	62500906	62506524	962	7	6	151	17,252.52	7.58	56.36-unst	57.62	−0.645	Nuclear
SbHP4	LOC8068945	9	55183674	55186538	858	6	5	154	17,682.91	6.14	56.83-unst	60.19	−0.678	Nuclear
SbHP5	LOC8055697	9	8016183	8018505	900	5	4	151	17,800.24	8.42	60.23-unst	72.32	−0.754	Nuclear
Response Regulators (RRs)														
Type-A RRs														
SbRR4	LOC8077366	3	73177403	73181260	1873	5	4	245	26,365.79	5.24	51.26-unst	83.63	−0.41	Nuclear
SbRR9	LOC110435576	5	2740967	2743306	1,146	5	4	202	22,749.54	6.13	59.58-unst	79.11	−0.683	Nuclear
SbRR10	LOC8071408	8	1026753	1029205	1,304	5	4	279	30,788.82	6.56	69.03-unst	83.55	−0.352	Nuclear
Type-B RRs														
SbRR16	LOC8056131	3	70434838	70438749	2055	5	4	579	64,700.22	5.04	45.88-unst	81.3	−0.424	Nuclear
SbRR17	LOC8079408	4	5416832	5421301	2579	6	5	631	68,631.82	6.12	39.97	77.4	−0.533	Nuclear
SbRR18	LOC8084640	4	66411416	66416077	3155	6	5	675	73,136.19	5.96	46.1-unst	80.99	−0.341	Nuclear
SbRR19	LOC8056866	1	72774008	72779535	2724	6	5	686	73,679.76	6.26	49.94-unst	77.76	−0.423	Nuclear
SbRR20	LOC8076203	10	5132133	5136115	2344	6	5	672	72,886.35	6.01	46.69-unst	80.45	−0.331	Nuclear
SbRR24.1	LOC8058416	10	55120219	55130513	2088	8	7	695	76,440.93	5.56	43.44-unst	74.46	−0.459	Nuclear
SbRR24.2	LOC8084794	8	9453701	9488726	2163	9	8	551	60,852.22	5.66	36.22	80.69	−0.536	Nuclear
Type-C RRs														
SbRR13	LOC8083419	6	3752870	3753702	501	2	1	132	14,169.76	7.74	18.5	96.74	0.224	Cytoplasmic
SbRR14	LOC8085961	1	7312249	7313602	963	4	3	201	21,501.8	9.69	54.67-unst	88.41	−0.106	Chloroplast
Pseudo-RRs														
SbPRR1	LOC8072479	4	56625637	56628735	2328	6	5	524	58,166	5.91	52.76-unst	66.37	−0.623	Nuclear
SbPRR2.1	LOC8078076	3	302694	310420	2682	6	5	395	42,195.43	6.06	56.94-unst	68.3	−0.385	Nuclear
SbPRR2.2	LOC8057890	10	8684841	8687455	1952	6	5	466	50,071.47	5.01	56.48-unst	68	−0.438	Nuclear
SbPRR3.1	LOC8070845	6	40305107	40316802	3093	10	9	291	31,977.47	5.08	55.20-unst	70.34	−0.646	Nuclear
SbPRR3.2	LOC8084889	1	69433326	69440358	3195	12	11	765	83,595.91	6	52.17-unst	59.52	−0.88	Nuclear
SbPRR5	LOC110432809	2	65784791	65790044	2553	8	7	630	70,207.3	6.2	52.28-unst	65.3	−0.827	Nuclear
SbPRR9	LOC8058579	5	4190668	4195535	2582	8	7	697	75,487.93	7.32	56.89-unst	62.48	−0.716	Nuclear

### 3.6 Phylogenetic Analysis

**FIGURE 2:**
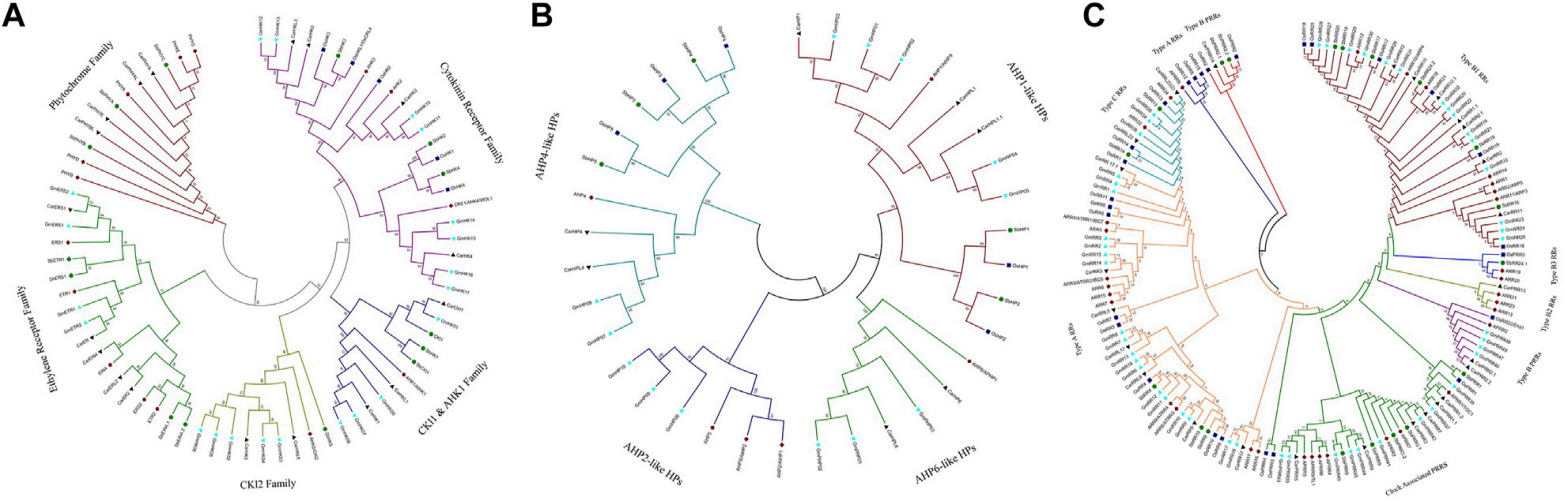
Phylogenetic analysis of **(A)** HK, **(B)** HPs **(C)** of RR and PRRs proteins in *S. bicolor, O. sativa, A. thaliana, G.* max and *C. arietinum*. The bracket indicates the relative divergence of examined sequences. Neighbour-joining method with bootstrap analysis (1,000 replicates) was used to drive tree from the alignment of protein sequences. Evolutionary analysis was conducted in MEGA7 (Kumar et al., 2016).

This study aimed to look at the evolutionary pattern and phylogenetic relationship of TCS proteins in *S. bicolor*; a neighbor-joining tree was constructed using the full-length protein sequence alignment of the identified TCS proteins from *S. bicolor*, *A. thaliana*, *G. max*, *O. sativa*, and *C. aritenum* (Figure 2)*.* These proteins were divided into three groups: RRs, phosphotransfer proteins (HPs), and HKs. HKs were divided into five subgroups (CKl2, HK1 and CKl1, ethylene receptor, cytokinin receptor, and phytochrome families) according to the phylogenetic tree. Three cytokinin receptors were present in *A. thaliana* (*AHK2*, *AHK3*, and *AHK4*), as well as in *S. bicolor* (*SbHK2*, *SbHK3*, and *SbHK4*). Phylogenetic analysis revealed that *SbHK3* and *SbHK4* were orthologues of *AHKs*. Cytokinin receptors in *A. thaliana* have been functionally characterized and shown to control a variety of cytokinin-regulated processes, such as leaf senescence, stress responses, seed size, vascular differentiation, and cell division. Based on phylogenetic analysis, it can be suggested that the predicted cytokinin receptors of *S. bicolor* have similar functions as their *A. thaliana* counterparts.

*AHK1* is a transmembrane protein which plays a role in osmosensing. It is largely expressed in the roots of *A. thaliana* in salt stress conditions. In *S. bicolor*, *SbHK1* and *SbCKl1* are the two orthologues of *AHK1*. In the CKl2 group, phylogenetic analysis indicated that *SbHK5* is an orthologue of *AHK5*/*ACKl2*. The ethylene receptors *SbETR1* and *SbERS1* are present as direct homologs of *ERS1* and *ETR1*. Similarly, *SbEIN4.1* and *SbEIN4.2* proteins are present on another clade, making a direct orthologue relationship with *A. thaliana ETR2* and *ERS2*.

Phytochromes or photoreceptors are found to be involved in growth and development in light stress conditions. *A. thaliana* contains five photoreceptors*.* These contain a C-terminal, a GAF domain, an N-terminal PHY domain, one HisKa, and two PAS domains, which are involved in signal transduction pathways. Three photoreceptors (*SbPHYA*, *SbPHYA*, and *SbPHYC*) were identified in *S. bicolor*, with the same domains as those present in *A. thaliana.* Five HPs were found in *S. bicolor*, showing close phylogenetic relationships to the true HPs of *A. thaliana* and *O. sativa*. These HPs were grouped according to their phylogenetic relationships with their *A. thaliana* counterparts. *SbHP1* and *SbHP2* were grouped as being AHP1-like. *SbHP3*, *SbHP4*, and *SbHP5* were grouped as being AHP4-like HPs.

Protein sequences of *S. bicolor*, *O. sativa*, *A. thaliana*, *G. max*, and *C. Arietinum* were used for the phylogenetic analysis of RRs. RRs are classified as PRRs (divergent RRs), type-A RRs, type-B RRs, or type-C RRs. This third family of TCS genes regulates the final responses to environmental stresses. PRRs are not considered to be true RRs due to the absence of a DDK conserved motif. It was confirmed that *SbRRs* are true RRs, since they have close phylogenetic relationships with their *A. thaliana* and *O. Sativa* counterparts. The evolution pattern of this family in *S. bicolor* revealed that this is segmentally duplicated. The same results have been found in *O. sativa* and *A. thaliana* in which it is also segmentally duplicated. Because type-A RRs are not found in unicellular algae and are observed only in land plants, they are considered relatively new members of the RR family and have been suggested to perform some novel functions in those organisms. Their structure contains a Rec domain and a conserved DDK motif that is important for receiving the phosphate group. Mainly, cytokinin activates type-A RRs, and this cytokinin induction partially depends on type-B RRs.

### 3.7 Gene Structure and Conserved Motif Analysis

**FIGURE 3:**
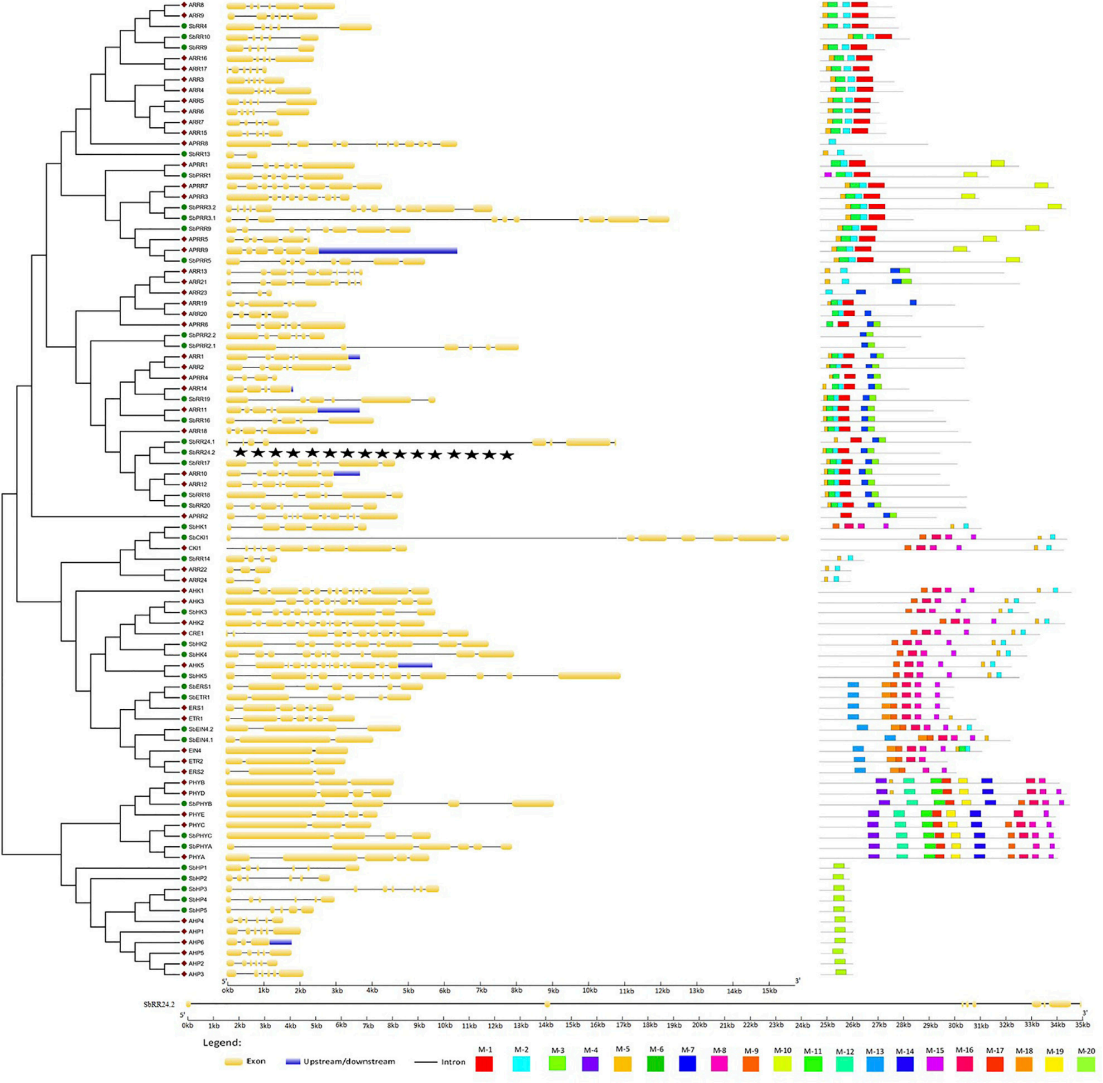
Gene structure and conserved motifs of all HK(L), HP and RR family genes in *S. bicolor.* In gene structure, the exons, introns, and untranslated regions are indicated by yellow boxes, black lines and blue boxes, respectively. In conserved motifs, different color represents different motifs.

A crucial evolutionary feature of a gene is its exon-intron structure and it provides information about its functional diversity. Therefore, the exon-intron organization of the *AtTCS* and *SbTCS* genes was further analyzed (Figure 3). The results showed that the members of the cytokinin receptor (HK) family had exon numbers ranging from 5 in *SbHK1* to 14 in *SbHK5* with introns 4–13. The *A. thaliana* HK family has exons 11–14 and introns 10–13. *SbCKl1* was found to have 8 exons and 7 introns, whereas *AtCKl1* contains 9 exons and 9 introns. Ethylene receptor family members *SbEIN4.1* and *SbEIN4.2* were shown to contain 3 exons and 2 introns, whereas *SbERS1* and *SbETR1* contained 6 exons and 5 introns. *A. thaliana ERS1* contains 6 exons and 5 introns, *ETR1* contains 7 exons and 6 introns, and *AtEIN4*, *ETR2*, and *ERS2* have 2–3 exons and 1–2 introns. In the phytochrome family members, *SbPHYA* was found to have 6 exons and 5 introns. *SbPHYB* and *SbPHYC* were both shown to have 4 exons. The HP family members have 5–7 exons and 4–6 introns. *A. thaliana* HP family members have 3–5 exons and 2–4 introns.

The *S. bicolor* RR family genes had a number of exons varying from 2 to 12 and an intron number varying from 1 to 11. The maximum number of exons and introns in *SbPRR3.2* was found to be 12 and 11, respectively. The same number of exons and introns were found in *AtRRs.* These results showed that the groups and members with closer phylogenetic relationships contain a similar exon-intron structure. Subsequently, we used the MEME software to predict the conserved motifs of these *TCS* genes (Figure 5). The overall number of identified motifs was 20. Motifs 2, 8, 15, 16, 17, and 18 were conserved in the whole cytokinin receptor family of *S. bicolor* and *A. thaliana* . The same motifs were present in *SbCKl1* and *CKl1.* The members of the ethylene receptor sub-family also contain similar motifs to those of the cytokinin receptor family but with the additional conserved motif 13; *SbERS1* and *SbETR1* lacked motifs 2 and 18. The *S. bicolor* and *A. thaliana* phytochrome family members contain an average of 10 conserved motifs (1, 4, 9, 11, 12, 14, 19, 15, 16, and 17). Only motif 20 is conserved in the members of the HP family.

Motifs 1, 2, 3, and 5 were conserved in all members of the type-A RR family. Type-B RRs contain the same motifs as type-A RRs and contain two more conserved motifs: motifs 6 and 7. The same motifs were present in two type-C RR members (*SbRR24.1* and *SbRR24.2*). *SbRR13* and *SbRR14* encoded only two conserved motifs (motifs 2 and 5). The members of the PRRs family contain the same set of motifs as type-A RRs, except for two members, which contain only two motifs (motifs 6 and 7). The members of the same gene families share the same motifs, indicating that there is no significant functional and sequence divergence between them. Collectively evolutionary analysis revealed that TCS is conserved.

### 3.8 Genomic Distribution, Gene Duplication, and Synteny Analysis of the *Sorghum bicolor* Two-Component System Members

**FIGURE 4:**
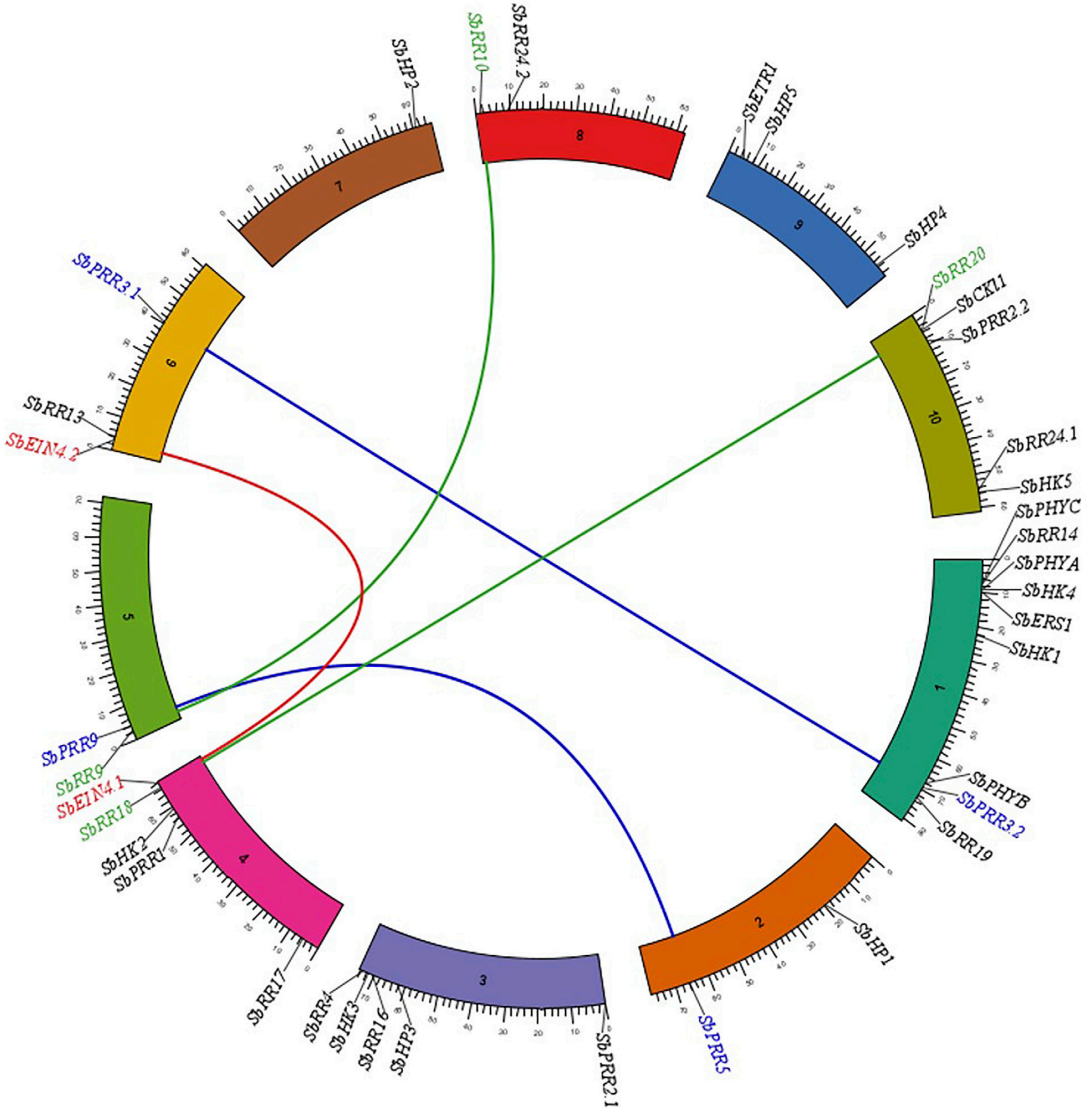
Syntenic analysis of TCS family genes in *S. bicolor*. The genes on different circular bar-blocks indicate the chromosomal position of genes. Green, blue and red color lines represent the duplicated pairs.

To examine the genomic distribution of the *SbTCS* genes, their chromosome gene location and duplication events were identified using syntenic analysis. All the identified *S. bicolor* TCS family members were found to be distributed on 10 chromosomes (Figure 4). These genes are unevenly distributed, since chr1 contains 9 (maximum number) genes, while chromosome 7 contains only one gene. The HK(L)s are randomly distributed on all *S. bicolor* chromosomes, except for chr2, chr5, chr7, and chr8. The members of the HP family are located on chr2, chr3, chr7, and chr9. RR family members are distributed on all chromosomes, except for chr7 and chr9.

The duplication events were analyzed for the *SbTCS* gene family. Since gene duplication provides raw material for development, the evolution of new genes in the genome was also analyzed. The number of tandem and segmental duplication events was observed to increase in a number of plant genes. When identifying the potential genomic duplication events, five pairs of TCS syntenic paralogs were found in the *S. bicolor* genome. This indicated that the *SbTCSs* have a high gene family expansion (Figure 6). In this study, the duplicated pairs resulting from segmental duplication include *SbPRR3.1/SbPRR3.2*, *SbPRR5/SbPRR9*, *SbEIN4.1/SbEIN4.2*, *SbRR18/SbRR20*, and *SbRR9/SbRR10.* In *S. bicolor*, multiple pairs exhibited segmental duplication, implying that the expansion of *SbTCS* genes is mainly due to segmental duplication. A similar expansion pattern exists in other plants, such as in *G.* max (Mochida et al., 2010), *A. thaliana* (Hutchison and Kieber, 2002), and Chinese cabbage (Liu et al., 2014). The synonymous rate (*Ks*), non-synonymous rate (*Ka*), and the *Ka/Ks* ratio of these duplications were calculated, and the *Ks* values were used to speculate on the duplication time (Table 3). The *Ks* of five segment duplicates ranged from 0.143 to 0.632. Therefore, the divergent time ranged from 10.89939024 to 48.17073171 Mya.

**TABLE 3:** Ks, Ka and Ka/Ks calculation and divergence time of the duplicated *SbTCS* gene pairs.

Duplicated gene pairs	Ks	Ka	Ka/Ks	Time (MYA^a^)	Duplication type
SbEIN4.1/SbEIN4.2	0.3299	0.4709	1.427402243	25.14481707	Segmental
SbRR9/SbRR10	0.143	0.1004	0.702097902	10.89939024	Segmental
SbRR18/SbRR20	0.1715	0.2077	1.211078717	13.07164634	Segmental
SbPRR3.1/SbPRR3.2	0.2885	0.2357	0.816984402	21.98932927	Segmental
SbPRR5/SbPRR9	0.632	0.8932	1.413291139	48.17073171	Segmental

a MYA: million years ago.

### 3.9 Promoter Analysis of the *SbTCS* Genes

**FIGURE 5:**
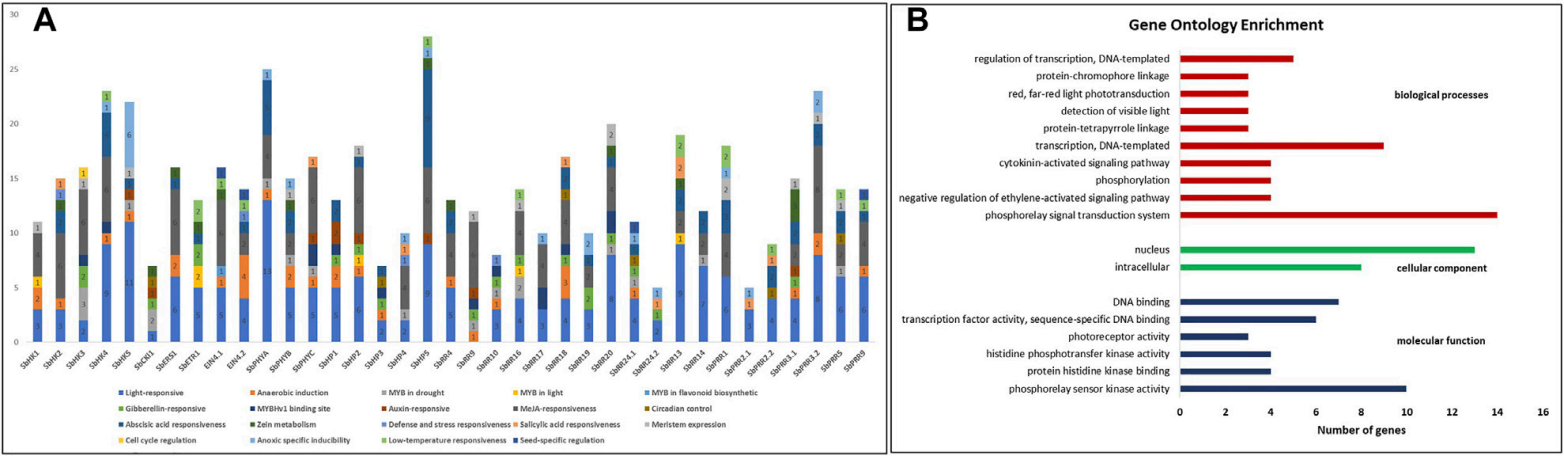
**(A)** Graphical representation of *cis*-regulatory elements presents in putative TCS promoter’s region. Different colors are representing different cis-elements and numbers in bars represent number of elements. **(B)** Gene ontology enrichment statistics graph, red color bar represents biological processes, green color bar represents cellular component, and blue color bar represent molecular function.

For a better understanding of the transcriptional regulation and functional role of the *SbTCS* genes, their promoter sequences were investigated to predict the *cis*-regulatory elements. Several hormone-related and abiotic stress-related *cis*-regulatory elements were identified, of which TATA- and CAAT-box were present in almost all of the 37 *SbTCSs* (Figure 5A; Supplementary Table S3)*.* Among them, the methyl jasmonate (meJa)-responsiveness elements were found in 27 *SbTCSs.* The ABA-responsive element (ABRE) involved in the abscisic acid response was present in 23 *SbTCSs.* A large number of *cis-*regulatory elements were associated with light signaling, including the GATA motif, ACE, box 4, G-box, and the TCCC and TCT motifs. Gibberellin-responsive elements (GARE motif, TATC-box, and P-box) were present in almost 10 *SbTCSs.* Auxin-responsive elements, such as AuxRR-core and TGA-element, and salicylic acid-responsive elements (TCA element and SARE) were also found. In addition, low temperature-responsiveness (LTR) elements were present in 11 *SbTCSs*, and drought-inducibility element (MBS) was found in 14 *SbTCSs.* These results demonstrate that the *TCS* genes are potentially involved in growth and developmental processes related to hormone metabolism and signal transduction networks. The presence of LTRs, TC-rich repeats (defense-responsive element), and MBS (MYB binding site), which are associated with drought-inducibility, suggest that TCS plays a vital role in the development of plant and multiple abiotic stress responses.

### 3.10 Gene Ontology Enrichment Analysis of *SbTCS* Genes

Gene ontology enrichment analysis of all the *SbTCS* genes was performed. For this purpose, a well-known open-source DAVID bioinformatics resource 6.7 was used. All the identified genes were subjected to DAVID gene ontology analysis by using the entrez_gene_id. The results revealed the identified genes were classified into three main functional biological categories annotated by GO, that including molecular function, cellular component and biological process (Figure 5B). In the category of biological processes 23 out of 37 genes were found to be involved and the highest proportion (14 out of 23) was found in the phosphorelay signal transduction system. Moreover 9 out of 23 genes were found to be involved in transcription, DNA-templated. In the category of molecular function 21 out of 37 genes were found to be involved in different functions, including phosphorelay sensor kinase activity (10), protein histidine kinase binding (4), histidine phosphotransfer kinase activity (4), photoreceptor activity (3), transcription factor activity; sequence-specific DNA binding (6), and DNA binding (7). Lastly in the category of cellular component, 25 out of 37 genes were found to be involved in two components including intracellular and nuclear.

### 3.11 Expression Analysis of the *SbTCS* Genes

**FIGURE 6:**
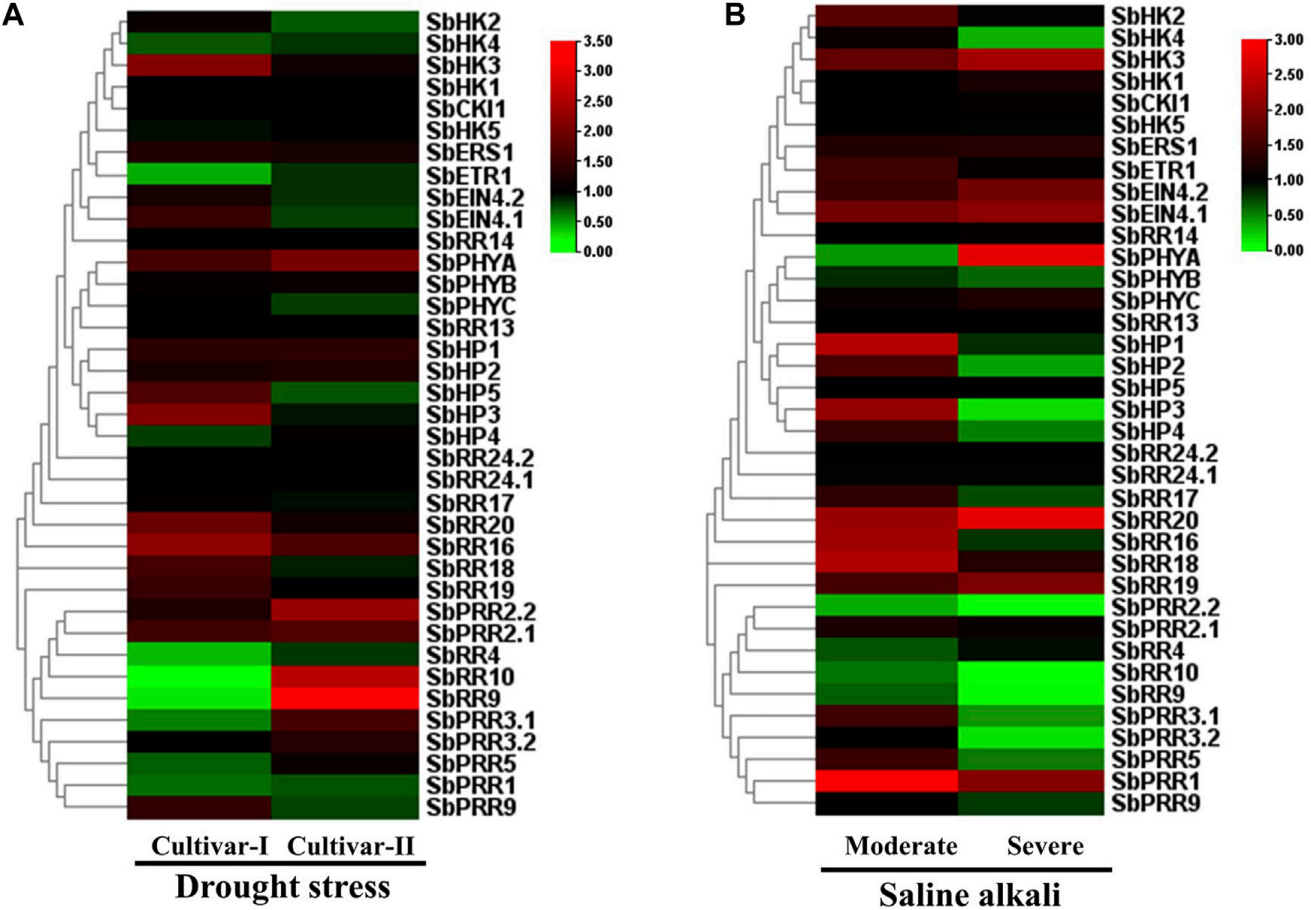
Heat map representing the response patterns of *SbTCS* genes under drought **(A)** and salt **(B)** stresses. Red color represents up-regulation, green color represents down-regulation and black color represent there is no change in expression.

To examine the expression level of the 37 *SbTCS* genes under different abiotic stress conditions, the publicly available RNA-seq data of *S. bicolor* was obtained from the SRA-NCBI database. The results showed that *SbHK3*, *SbPHYA*, *SbHP3*, and *SbHP5* were upregulated under drought stress in leaves. Most members of the RR family were upregulated under drought stress, whereas the expression levels of *SbETR1*, *SbRR4*, *SbRR10*, *SbRR9*, *SbRR3.1*, *SbPRR5*, *SbPRR1*, and *SbPRR9* were decreased (Figure 6A).

In the salt treatment, many HK family members were showed to be upregulated, including *SbPHYA*, *SbEIN4*.1, and *SbEIN4*.1. Almost all members of the HP family were upregulated during salt stress conditions. The RR family members, including *SbRR20*, *SbRR16*, *SbRR18*, *SbRR19*, and *SbPRR1*, were also upregulated. There were no expression changes observed in *SbHP3*, *SbPRR2.2*, *SbRR10*, *SbRR9*, and *SbPRR3.2* (Figure 6B). These results indicate that the *S. bicolor TCS* genes potentially play a crucial role in diverse abiotic responses.

### 3.12 Expression Validation of the *SbTCS* Genes Through qRT-PCR

**FIGURE 7:**
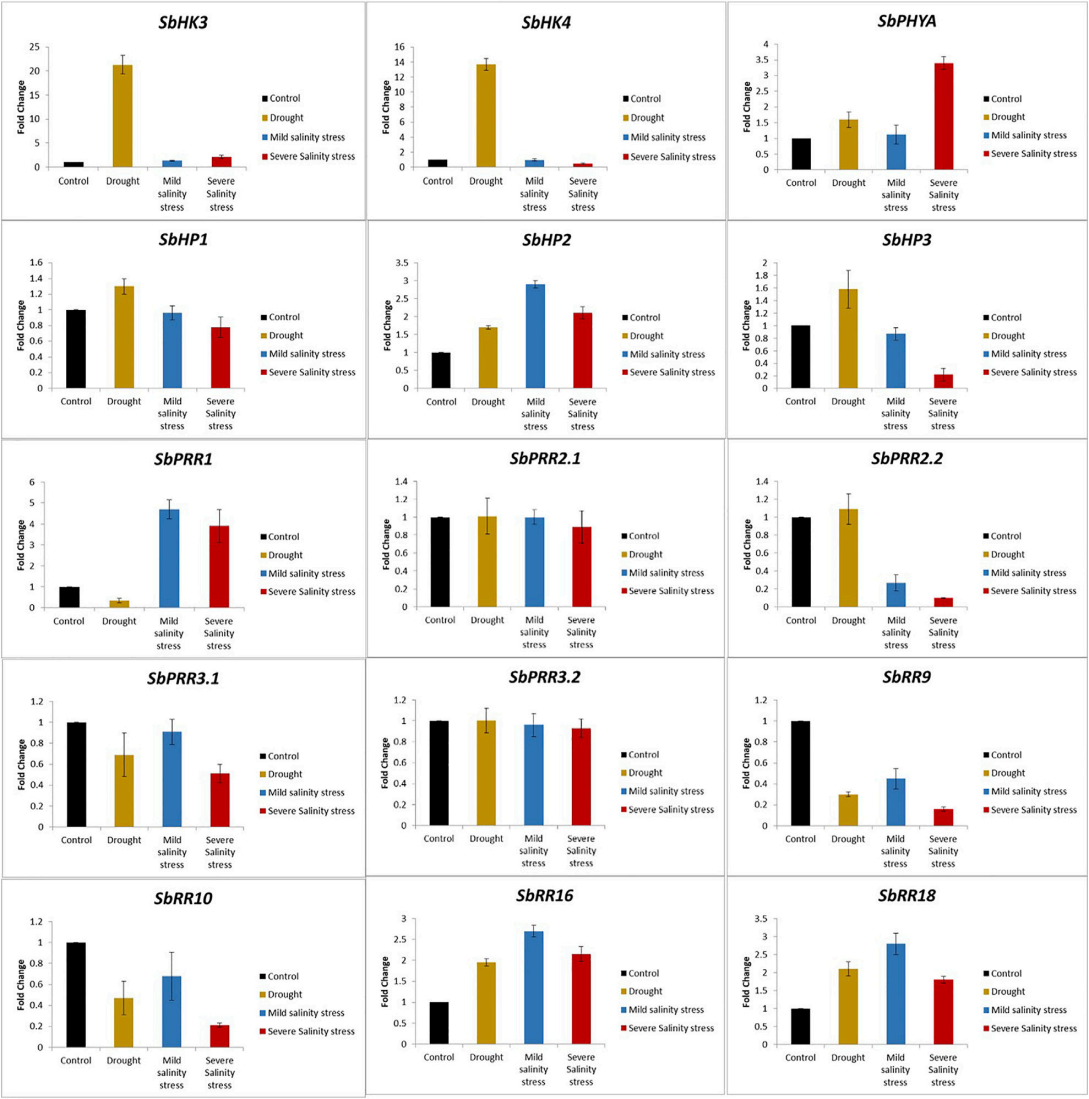
Expression profiling of TCS genes in response to drought (Yellow), mild salinity (Blue) and severe salinity (Red) stresses. Data represent means (SD) of three biological replicates. Vertical bars indicate standard deviations.

To further endorse the expression of *SbTCS* genes, 15 differentially expressed *SbTCS* genes based on RNA-seq analysis (belonging to different groups) were selected. The findings revealed that the overall expression trend of these genes obtained through qRT-PCR analysis was highly consistent with the RNA-seq data (Figure 7). Furthermore, compared with the control, the results revealed that drought stress treatment enhanced the expression of the *SbHK3*, *SbPHYA*, *SbHP1*, *SbHP2*, *SbHP3*, *SbPR2.2*, *SbRR16*, and *SbRR18* genes up to several folds higher. Meanwhile, compared with the control, drought stress decreased the expression of the *SbHK4*, *SbPRR1*, *SbPRR3.1*, *SbRR9*, and *SbRR10* genes. Drought stress seems to not have affected the expression of *SbPRR2.1* and *SbPRR3.2*.

Additionally, *SbHK3*, *SbHP2*, *SbPRR1*, *SbRR16*, and *SbRR18* were shown to be significantly highly expressed and *SbHP3*, *SbPRR2.2*, *SbPRR3.1*, *SbRR9*, and *SbRR10* were shown to not be significantly expressed in both mild and severe salt stress conditions. No expression changes were observed for *SbPRR2.1* and *SbPRR3.2* under salt stress conditions. *SbPHYA* and *SbPRR1* were found to be significantly overexpressed, by up to three-fold, under salt stress conditions. All of these findings show the potential functional importance of *TCS* genes in the growth and development of *S. bicolor* under different abiotic stresses.

## 4 Discussion

Plants are subjected to a variety of environmental factors that can inhibit their growth, development, and yield. Plants cannot avoid these conditions since they are sessile; thus, they have developed multiple signaling cascades to survive. The *TCS* gene family has a vital role in the signal transduction pathway and, thus, in plant growth and development. As a result, the identification and functional validation of TCS in signal transduction and metabolic pathways may aid in developing crops with improved traits, such as stress tolerance, to meet global climate change challenges. These investigations have been carried out in a variety of model and non-model plant species. In tomato, ethylene receptors have a role in pollen thermotolerance (Firon et al., 2012). Elements from *A. thaliana* TCS can play a role in phosphorelay interaction in physiologically irrelevant fungal systems (Lohrmann and Harter 2002). In *G. max*, these genes are expressed under dehydration stress (Le et al., 2011). However, little is known about this gene family’s diversity in *S. bicolor.* In this research, a genome-wide investigation of the TCS gene family was carried out in *S. bicolor*, including the gene structure, conserved motifs, sequence phylogeny, and chromosomal localization. RNA-seq and qRT-PCR expression analyses of the SbTCS members was examined *in silico* under drought and salt stress.

The *TCS* gene family has been identified in various species of plants, including *A. thaliana* (Hwang 2002), *C. arietinum* (Ahmad et al., 2020), banana (Dhar et al., 2019), melon (Liu et al., 2020), cucumber, watermelon (He et al., 2016b), Chinese cabbage (Liu et al., 2014), *O. sativa* (Du et al., 2007), tomato (He et al., 2016a), and *G. max* (Mochida et al., 2010). In the *S. bicolor* genome, 37 TCS genes were identified in the present study. This number is the same as the number of members present in *O. sativa L*. (37) and is lower than that of *A. thaliana* (47), *L. japonicus* (40), *G. max* (98), *S. lycopersicum* (tomato) (65), *T. aestivum* (62), *Zizania latifolia* (69), and *P. trichocarpa* (49) (Table 1). *SbTCSs* are segmentally distributed on chromosomes. Four pairs of duplicated genes were found. Both tandem and segmental duplication were observed in the genome of several plants, such as melon, *Z. latifolia*, *A. thaliana*, and Chinese cabbage. The Chinese cabbage genome underwent whole genome duplication after diverging from *A. thaliana*; and its TCS genes originated mainly from segmental duplication (Liu et al., 2014). Similarly, nineteen duplicated genes were observed in *Z. latifolia* (He et al., 2020)*.* In cucumber, one event of tandem duplication has been found. In watermelon, one tandem and two segmentally duplicated gene pairs have been identified. This indicates that genome duplication plays an important role in the duplication of this gene family.

In *S. bicolor*, five pairs of segment duplicates, including *SbEIN4.1/SbEIN4.2*, *SbRR9/SbRR10*, *SbRR18/SbRR20*, *SbPRR3.1/SbPRR3.2*, and *SbPRR5/SbPRR9*, were found, which expanded the TCS gene family in *S. bicolor.* Similarly, in *A. thaliana*, *C. arietinum*, and Chinese cabbage, the main TCS gene duplication mechanism was segmental duplication. In 35.71% of all *A. thaliana* species, 10 pairs of TCS genes were found to be segmentally duplicated. In *C. arietinum*, 55.55% of the genes were involved segmentally duplicated. In Chinese cabbage, 61 of the 85 identified TCS genes were found to be duplicated as a result of segmental duplication. Meanwhile, in tomato, both segmental and tandem duplication events were identified (He et al., 2016a; Ahmad et al., 2020). In this study, the Ks for segmental duplication ranged from 0.143 to 0.632, which corresponds to a divergence time from 10.9 to 48.17 Mya. In tomato, the Ks for segmental duplication ranged from 0.6 to 0.79 with the divergence time ranging from 46 to 60 Mya and tandem the duplication time ranging from 5.96 to 26.55 Mya. In *C. arietinum*, the time of divergence for the first duplication event was 256.7 Mya and the latest duplication, which resulted in the production of a new gene, was a tandem duplication, and took place at about 38.90 Mya. This suggests that compared with segmental duplication, tandem duplication in plants occurred more recently since these replicates were more likely to regulate stress responses (Firon et al., 2012).

Phylogenetic analysis revealed the division of *SbTCS* genes into the same subfamilies as reported in previous studies of *A. thaliana* (Hutchison and Kieber, 2002), *S. lycopersicum* (Firon et al., 2012), and *Z. mays* (Chu et al., 2011). All these plants contain members from the three subfamilies: HK, HP, and RRs. The subfamilies in *S. bicolor*, as well as those in *A. thaliana*, were classified based on the conserved functional domains. In *A. thaliana, CKl1* was referred to as hybrid HK(L) due to its involvement in cytokinin signaling (Ishida et al., 2009). SbHK5 is a true HK and has been kept in the CKl2 group. The domains specifying the type RR family members in *A. thaliana* were the Rec/Response_reg domains. The clades of the RR family in *S. bicolor* had the same domains. In banana, *MaERS1.*A lacks the domain required for ethylene binding and signaling; therefore, it may not play a significant role in ethylene sensing (Dhar et al., 2019). In melon, no members of the type-C RR family have been found (Liu et al., 2020). In chickpea, the cytokinin receptors’ clade was clearly separated via phylogenetic analysis due to the presence of the CHASE domain. Conversely, various clades were revealed based on the phylogeny of RRs. Some members from one species clustered on same clade, while members of other plants clustered on another clade, such as the *O. sativa* clade (Type-B RRs), *A. thaliana* clade (Type-B3 RRs), and legume-specific clade (Type-B RRs) (Tiwari et al., 2021).

In the promoter of *SbTCS* genes, the analysis of the *cis*-elements helps to find the switches that are involved in regulating the transcription of downstream genes. Our result revealed the presence of several light-responsive, drought-responsive, and hormone-responsive elements relevant to stress and wounding responses. These elements have previously been found in the promoters of genes belonging to the *TCS* family in other plants. In banana, hormone- and light-responsive elements were abundant in the HK family. Hormone-responsive elements included TCA elements, ethylene- and gibberellin-responsive elements (ERE and GARE motif), and ABRE. Biotic and abiotic stress-responsive elements were also found in the RR family (Dhar et al., 2019). In cucumber and watermelon, a large number of similar stress responsive elements have been found, including ABRE, MBS, ABA-responsive, as well as drought-responsive ones (He et al., 2016b). In Chinese cabbage, apart from these stress-responsive elements, GARP binding sites are also present in type-A RRs. These promoters may act as a binding site to type-B ARRs, resulting in transcription stimulation. It has been shown that the induction of cytokinin-dependent type-A RRs is partly reliant on type-B RR transcriptional regulation (Liu et al., 2014). This suggests that the TCS genes are activated not only by hormonal stimuli, but also by other genes responding to stress or ripening conditions.

Abiotic factors such as drought, salt, and cold may have an impact on plant growth and development. TCSs play a role in controlling the plants’ response to abiotic stresses; thus, their expression patterns were examined to learn more about their involvement in coping with environmental changes. In *A. thaliana AHK1* is a positive regulator of drought and salt stress responses (Tran et al., 2007). *EIN2* of ethylene signaling and histidine kinase 5 may also be involved in regulating salt stress response (Lei et al., 2011; Pham et al., 2012). In current study, the *SbTCS* gene family was shown to have a tissue-specific expression. In leaves, *SbHK3*, *SbPHYA*, *SbHP3*, and *SbHP5* were upregulated under drought stress. *SbPHYA*, *SbEIN4*.1, and *SbEIN4*.1, HP family members, and RRs were also upregulated during high salinity conditions. Under drought stress, in Cultivar-I, a number of TCS genes were overexpressed. Almost similar results were observed in Cultivar-II. In moderate drought stress, RRs showed higher expression levels. Under severe drought stress, RRs were downregulated. In *C. arietinum*, *CarHK2*, *CarHK3*, *CarHKL3*, and *CarHK4* were expressed in all tissues, and *CarHK5* and *CarHK1* were expressed in pods and shoots. Members of the *CarRR* family were mostly expressed in flower buds (Ahmad et al., 2020). Similarly, *O. sativa* HK family members were expressed in the roots and leaves, HPs were expressed in leaves, and RRs were expressed in roots, leaves, stems, and spikelets (Du et al., 2007). Melon had the highest expression levels of RRs, indicating that these genes are important for root cytokinin signaling. Similar results were also observed in Chinese cabbage.

Among the abiotic stresses, drought stress (Azeem et al., 2019) and salt stress (Rasul et al., 2017) are the main threats to modern agriculture. TCS genes are involved in diverse abiotic stresses, according to mounting evidence. In this investigation, 37 *SbTCS* genes were identified, and various drought and salt stresses were shown to regulate these genes, some of which were downregulated, while most were upregulated in response. However, drought treatment induced the expression of 21 out of 37 genes. In *A. thaliana*, drought stress caused the downregulation of these genes, whereas in tomato, these stresses caused the upregulation of *SlHPs* and *SlRRs.* In *S. bicolor*, these genes were upregulated under drought stress. For example, *SbRR16*, *SbRR18*, *SbRR20*, and *SbPRR1* were upregulated and a few RRs showed negative expression but their *A. thaliana* counterparts, *ARR12* and *ARR1*, were downregulated in response to drought stress. In *C. arietinum,* Drought and salt stresses resulted in variable expression of these genes like *CarRR12* had lower expression in salt stress whereas, it given a higher expression in drought stress. Similarly, *CarRR2* also shown expression in drought stress. *CarRR17* had a higher expression in response to heat stress. A few genes including *CarRR5* and *CarRR12* were downregulated. In salt stress, *CarRR17*, *CarHK1, CarPHYA, CarHP1* and *CarERS1* had shown higher expression (Ahmad et al., 2020). In banana, ethylene treatment shown that *MaERS1.A, MaERS1.B* and *MaERS1.C* had higher expression (Dhar et al., 2019). Very few *SbTCS* genes showed a null expression. Similarly, in tomatoes, drought stress responses were confirmed to be modulated by *SlPHYA*, *SlPHB1*, and *SlPHB2.* These expression studies of TCS elements have crucial implications for how these genes function under abiotic stresses.

## 5 Conclusion

In this study, we identified a total of 37 putative members of the TCS protein family, which include 13 HK(L), 5 HPs, and 19 RRs. Protein classifications, phylogenetic relationships, gene structures, domains, chromosomal gene distribution, and gene duplication events were investigated in detail. These TCSs showed significant conservation of their sequence and domains. SbTCS proteins showed a closer phylogenetic relationship with the TCSs of other plants. Members of the SbRR family experienced significant gene duplication events, and segmental duplication resulted in the expansion of the genes. These findings provide important functional and regulatory information regarding the TCS genes of *S. bicolor*, which will help better understand the signal transduction pathways and improve the stress tolerance of this plant.

## Data Availability

All the sequence data used in the study were downloaded from the nucleotide repository of National Center for Biotechnology Information (NCBI; www.ncbi.nlm.nih.gov) and Ensemble plants database (http://plants.ensembl.org/index.html). RNA-seq data (BioProject: PRJNA319738 for drought stress tolerance and BioProject: PRJNA591555 for saline/alkali stress) was downloaded from NCBI Sequence Read Archive (SRA) database (https://www.ncbi.nlm.nih.gov/sra). The genome and gtf files were downloaded from NCBI Genome assembly (https://www.ncbi.nlm.nih.gov/assembly/GCF_000003195.3/). The other data generated in the study were included in this published article and its Supplemental Materials.
