# Supplementary material for: Two-Component System Genes in Sorghum bicolor: Genome-Wide Identification and Expression Profiling in Response to Environmental Stresses
Source: Front Genet. 2021 Nov 25;12:794305. doi: 10.3389/fgene.2021.794305 (PMC8655132; doi:10.3389/fgene.2021.794305)
Supplement: Supplementary file 1 [file DataSheet1.docx]

**Supplementary files:**

**Table S1:** List of primers used in this study

| **Locus ID** | **Gene Name** | **Primer Sequence** |
| --- | --- | --- |
| LOC110433965 | SbHK3F | CACCTCCCCTCGCAAGATTT |
|  | SbHK3R | AGTGGATGGATAGCCCCTGT |
| LOC8060004 | SbHK4F | TTTGCTTGCACCGACAGTTC |
|  | SbHK4R | CTTGGCTTGGGCATCCAATG |
| LOC8059991 | SbPHYAF | ATGGGGATTCCACTGGCTTG |
|  | SbPHYAR | GCTTTGCACCTCCCCATTTG |
| LOC8062536 | SbHP1F | CGACAGGATCATCTCCGAGC |
|  | SbHP1R | CCGCATAGGGCAGAAGGTAG |
| LOC8057993 | SbHP2F | CAAAATCCACGACGGGCTTG |
|  | SbHP2R | TCGTGTGGAATTCGGGTTGT |
| LOC8075559 | SbHP3F | GCGCCTTTCGCCATCTTTTT |
|  | SbHP3R | GTGGGTACCGACCAATCGAG |
| LOC8072479 | SbPRR1F | CCAGCCTCTCCGGGAAGAA |
|  | SbPRR1R | CCATAGGGTACTGCACAGGC |
| LOC8078076 | SbPRR2.1F | GATGTGGACGACAACCTGCT |
|  | SbPRR2.1R | GCTTCTCCTCTTGTGCTGCT |
| LOC8057890 | SbPRR2.2F | CGAGGAGGACAACGTCACAA |
|  | SbPRR2.2R | CACATGCATGCGTAGTAGTGT |
| LOC8070845 | SbPRR3.1F | CCAAGGGGCAGAATGGAAGT |
|  | SbPRR3.1R | TAGCCTGAACCACAAACATGC |
| LOC8084889 | SbPRR3.2F | GCAATGGCCAGAATGGAACG |
|  | SbPRR3.2R | ACTACGACAGGCATGTAACTGA |
| LOC110435576 | SbRR9F | ATGGATCGCGCTAACCAGAG |
|  | SbRR9R | TGGCCATTCAATGACTAAAAGGG |
| LOC8071408 | SbRR10F | ACGCACAGAGACACACAGAG |
|  | SbRR10R | GTGCTTGGGTTTGTTGGCAT |
| LOC8056131 | SbRR16F | TTGTACTTAGGTCTTGATTGTCCT |
|  | SbRR16R | TCGGGAATGTTGGTCTGAGC |
| LOC8084640 | SbRR18F | GCTTTGTGTGCCATGCAGAA |
|  | SbRR18R | CCCTTGGAGCGCAAATGATG |
| House keeping gene  ( LOC110436299) | SbGAPDHF | AAGCTGAAGGTGGCGATCAA |
|  | SbGAPDHR | GCGATGACTTCCAGTGGTGA |

**Table S2: Domains Predictions of SbTCS Proteins**

| **Family** | **Gene Name** | **Gene ID** | **Pfam** | **SMART** | **CDD** |
| --- | --- | --- | --- | --- | --- |
| **Histidine Kinases (HKs)** | SbHK1 | LOC8059279 | HATPase_c, Response_reg, HisKA | HisKA, HATPase_c, REC | REC_hyHK_CKI1_RcsC-like, PRK11107 super family, HisKA |
|  | SbHK2 | LOC8075742 | CHASE, HATPase_c, Response_reg, HisKA | CHASE, HisKA, HATPase_c, REC | PRK11107 super family, CHASE |
|  | SbHK3 | LOC110433965 | CHASE, HATPase_c, Response_reg, HisKA | CHASE, HisKA, HATPase_c, REC | PRK11107 super family, CHASE |
|  | SbHK4 | LOC8060004 | CHASE, HATPase_c, Response_reg, HisKA | CHASE, HisKA, HATPase_c, REC | PRK11107 super family, CHASE |
|  | SbHK5 | LOC8069360 | HATPase_c, Response_reg, HisKA | HisKA, HATPase_c, REC | PRK11107 super family |
|  | SbCKl1 | LOC8076204 | HATPase_c, Response_reg | HisKA, HATPase_c, REC | REC_hyHK_CKI1_RcsC-like, PRK11107 super family, HisKA |
|  | SbERS1 | LOC8063415 | HATPase_c, GAF, HisKA | GAF, HisKA, HATPase_c | PRK11107 super family, GAF |
|  | SbETR1 | LOC8076011 | HATPase_c, GAF, HisKA | GAF, HisKA, HATPase_c | PRK11107 super family, GAF |
|  | SbEIN4.1 | LOC8155512 | GAF, Response_reg, HisKA | GAF, HisKA, HATPase_c, REC | HATPase_ETR2_ERS2-EIN4-like, REC super family, PRK11466 super family, GAF |
|  | SbEIN4.2 | LOC8066019 | Response_reg, GAF | GAF, HisKA, HATPase_c, REC | REC_ETR-like, HATPase_ETR2_ERS2-EIN4-like, GAF, HisKA |
|  | SbPHYA | LOC8059991 | PHY, PAS, PAS, PAS_2, GAF, HATPase_c, HisKA | GAF, PAS, PAS, HisKA, HATPase_c | COG4251 super family, HATPase super family, PAS, PAS, PRK15347 super family |
|  | SbPHYB | LOC8081072 | PHY, PAS, PAS, PAS_2, GAF, HisKA, HATPase_c | GAF, PAS, PAS, HisKA, HATPase_c | COG4251 super family, HATPase super family, PAS, PAS, BaeS |
|  | SbPHYC | LOC8086232 | PHY, PAS, PAS, PAS_2, GAF, HATPase_c, HisKA | GAF, PAS, PAS, PAC, HisKA, HATPase_c | COG4251 super family, HATPase_Phy-like, PRK15347 super family, PAS |
| **Phosphotransfer Proteins (HPs)** | SbHP1 | LOC8062536 | Hpt | HPT | Hpt |
|  | SbHP2 | LOC8057993 | Hpt | HPT | Hpt |
|  | SbHP3 | LOC8075559 | Hpt | - | Hpt |
|  | SbHP4 | LOC8068945 | Hpt | - | Hpt |
|  | SbHP5 | LOC8055697 | Hpt | - | Hpt |
| **Type-A Response Regulators (RRs)** | SbRR4 | LOC8077366 | Response_reg | REC | PLN03029 super family |
|  | SbRR9 | LOC110435576 | Response_reg | REC | PLN03029 super family |
|  | SbRR10 | LOC8071408 | Response_reg | REC | PLN03029 super family |
| **Type-B Response Regulators (RRs)** | SbRR16 | LOC8056131 | Response_reg, Myb_DNA-binding | REC | REC_typeB_ARR-like, myb_SHAQKYF, PLN03162 super family |
|  | SbRR17 | LOC8079408 | Response_reg, Myb_DNA-binding | REC | REC_typeB_ARR-like, myb_SHAQKYF, PLN03162 super family |
|  | SbRR18 | LOC8084640 | Response_reg, Myb_DNA-binding | REC | REC_typeB_ARR-like, myb_SHAQKYF |
|  | SbRR19 | LOC8056866 | Response_reg, Myb_DNA-binding | REC | REC_typeB_ARR-like, myb_SHAQKYF |
|  | SbRR20 | LOC8076203 | Response_reg, Myb_DNA-binding | REC | REC_typeB_ARR-like, myb_SHAQKYF |
|  | SbRR24.1 | LOC8058416 | Response_reg | REC | REC_typeB_ARR-like, myb_SHAQKYF |
|  | SbRR24.2 | LOC8084794 | Response_reg | REC | REC_typeB_ARR-like, myb_SHAQKYF, PLN03162 super family |
| **Type-c Response Regulators (RRs)** | SbRR13 | LOC8083419 | Response_reg | REC | REC_hyHK_CKI1_RcsC-like |
|  | SbRR14 | LOC8085961 | Response_reg | REC | REC_hyHK_CKI1_RcsC-like |
| **Pseudo-Response Regulators (PRRs)** | SbPRR1 | LOC8072479 | CCT, Response_reg | REC | REC super family, CCT |
|  | SbPRR2.1 | LOC8078076 | Myb_DNA-binding | - | PLN03162 super family |
|  | SbPRR2.2 | LOC8057890 | Myb_DNA-binding | - | PLN03162 super family |
|  | SbPRR3.1 | LOC8070845 | Response_reg | REC | REC super family |
|  | SbPRR3.2 | LOC8084889 | Response_reg, CCT | REC | REC super family, CCT |
|  | SbPRR5 | LOC110432809 | Response_reg, CCT | REC | psREC_PRR, CCT, PLN03029 super family |
|  | SbPRR9 | LOC8058579 | Response_reg, CCT | REC | REC super family, CCT |

**Table S3**: Promoter analysis of TCS genes.

| Regulatory Element | Core Seq. | SbHK1 | SbHK2 | SbHK3 | SbHK4 | SbHK5 | SbCKl1 | SbERS1 | SbETR1 | EIN4.1 | EIN4.2 | SbPHYA | SbPHYB | SbPHYC | SbHP1 | SbHP2 | SbHP3 | SbHP4 | SbHP5 | SbRR4 | SbRR9 | SbRR10 | SbRR16 | SbRR17 | SbRR18 | SbRR19 | SbRR20 | SbRR24.1 | SbRR24.2 | SbRR13 | SbRR14 | SbPRR1 | SbPRR2.1 | SbPRR2.2 | SbPRR3.1 | SbPRR3.2 | SbPRR5 | SbPRR9 | Function |
| --- | --- | --- | --- | --- | --- | --- | --- | --- | --- | --- | --- | --- | --- | --- | --- | --- | --- | --- | --- | --- | --- | --- | --- | --- | --- | --- | --- | --- | --- | --- | --- | --- | --- | --- | --- | --- | --- | --- | --- |
| **CAAT-box** | **CAAAT** | **4** | **8** | **8** | **3** | **4** | **8** | **7** | **5** | **6** | **9** | **6** | **2** | **3** | **4** | **4** | **5** | **3** | **1** | **5** | **9** | **6** | **3** | **6** | **7** | **4** | **1** | **3** | **6** | **3** | **5** | **1** | **6** | **6** | **7** | **7** | **2** | **3** | common cis-acting element in promoter and enhancer regions |
|  | **CAAT** |  |  |  |  |  |  |  |  |  | **1** | **6** | **1** |  |  |  |  |  |  |  |  |  |  |  |  |  |  |  |  |  |  |  |  |  |  |  |  |  |  |
|  | **CCAAT** |  | **3** |  |  |  |  | **2** | **1** |  |  | **2** |  |  | **3** |  | **2** | **3** |  |  |  | **2** | **5** | **1** | **3** | **2** | **2** | **2** |  | **1** | **2** |  | **2** | **2** | **4** | **2** |  |  |  |
|  | **AGCTCAATTTCA** |  |  |  |  |  |  |  |  |  |  |  |  |  |  |  |  |  |  |  |  |  |  | **1** |  |  |  |  |  |  |  |  |  |  |  |  |  |  |  |
|  | **CCCAATTT** |  |  |  |  |  |  |  |  |  |  |  |  |  |  |  |  |  |  |  |  |  |  |  | **1** | **1** |  |  |  |  |  |  |  |  |  |  |  |  |  |
|  | **TGCCAAC** |  |  | **1** |  |  |  |  |  |  |  |  | **2** |  | **2** |  |  |  |  |  |  |  |  |  | **1** |  |  |  |  |  |  |  |  |  |  |  |  |  |  |
|  | **CAACCAACTCC** |  |  |  |  | **1** |  |  |  |  |  |  |  |  |  | **1** |  |  |  |  |  |  |  |  |  |  |  |  |  |  |  |  |  |  |  |  |  |  |  |
| **GARE-motif** | **TCTGTTG** |  |  | **1** |  |  | **1** |  |  |  |  |  |  |  |  |  |  |  |  |  |  |  |  |  |  | **1** |  |  |  |  |  |  |  |  |  |  |  |  | gibberellin-responsive element |
| **GATA-motif** | **AAGGATAAGG** |  |  |  |  | **1** | **1** |  |  |  |  | **1** |  |  |  | **1** |  |  |  |  |  |  | **1** |  |  | **1** |  |  |  | **1** |  |  |  |  |  |  |  |  | part of a light responsive element |
|  | **GATAGGA** |  |  |  |  |  |  |  |  |  | **1** |  |  |  |  |  |  |  |  |  |  |  |  |  |  |  |  |  |  |  |  |  |  |  |  |  |  |  |  |
|  | **AAGATAAGATT** |  |  |  |  |  |  |  |  |  |  |  |  |  |  |  |  |  |  |  |  |  |  |  |  |  |  |  |  | **1** |  |  |  |  |  |  |  |  |  |
| **MBS** | **CAACTG** |  |  | **3** |  | **1** | **2** |  |  |  |  | **1** | **1** | **1** |  |  |  | **1** |  |  | **1** | **1** | **2** |  |  |  | **1** | **1** |  |  | **1** |  |  |  |  |  | **1** |  | MYB binding site involved in drought-inducibility |
| **O2-site** | **GATGATGTGG** |  |  |  |  |  |  |  |  |  |  |  | **1** |  |  |  |  |  | **1** | **1** |  |  |  |  |  |  | **1** |  |  | **1** |  |  |  |  | **3** |  |  |  | cis-acting regulatory element involved in zein metabolism regulation |
|  | **GATGA(C/T)(A/G)TG(A/G)** | | | |  |  | **1** |  | **1** |  |  |  |  |  |  |  |  |  |  |  |  |  |  |  |  |  |  |  |  |  |  |  |  |  |  |  |  |  |  |
|  | **GTTGACGTGA** |  | **1** |  |  |  |  | **1** |  | **1** |  |  |  |  |  |  |  |  |  |  |  |  |  |  |  |  |  |  |  |  |  |  |  |  |  |  |  |  |  |
| **TATA-box** | **TATAA** | **3** | **2** |  |  | **3** | **2** | **2** | **6** | **3** | **2** | **1** |  | **2** | **3** | **2** | **4** | **4** |  | **2** |  |  | **2** | **1** |  | **1** |  | **1** | **2** | **1** | **2** |  | **4** |  | **1** | **1** | **2** | **3** | core promoter element around -30 of transcription start |
|  | **TATA** | **8** | **10** | **3** | **1** | **8** | **2** | **5** | **13** | **8** | **10** | **5** | **1** | **6** | **13** | **3** | **7** | **8** |  | **7** | **1** | **2** | **10** | **1** | **4** | **1** | **1** | **6** | **2** | **3** | **7** | **1** | **20** | **9** | **9** | **8** | **3** | **8** |  |
|  | **ATTATA** | **1** | **1** |  |  |  | **1** | **1** | **5** |  |  | **1** |  | **1** | **3** | **1** |  |  |  | **2** |  |  |  |  |  | **1** |  |  |  |  | **1** |  | **1** |  | **1** |  |  |  |  |
|  | **TATAAAT** |  | **1** |  |  |  |  |  | **1** |  |  |  |  |  |  | **1** |  | **1** |  |  |  |  |  |  |  |  |  | **1** |  | **1** |  |  | **2** |  |  |  |  | **2** |  |
|  | **TATAAA** | **1** | **1** |  |  | **3** |  | **1** |  | **2** | **2** |  |  |  |  | **1** | **3** | **1** |  |  |  |  |  | **1** |  |  |  | **1** | **2** | **1** |  |  | **5** |  |  |  |  | **2** |  |
|  | **ATATAA** |  | **1** | **2** |  | **1** |  |  | **3** |  |  |  |  |  | **3** |  |  | **2** |  | **1** |  | **1** |  |  |  |  |  | **1** |  |  | **1** |  | **4** |  |  | **3** |  | **1** |  |
|  | **TATATA** | **1** | **1** | **1** |  | **2** |  | **2** | **1** | **4** | **1** |  |  |  | **7** |  |  |  |  | **2** |  |  | **4** |  | **2** |  |  | **1** |  | **1** |  |  | **7** | **2** | **3** | **1** |  | **1** |  |
|  | **ATATAT** | **4** | **2** |  |  | **3** |  | **3** | **3** | **4** | **1** |  |  |  | **6** |  | **1** | **1** |  | **1** |  |  | **5** |  | **1** |  |  | **1** |  | **3** | **1** |  | **5** | **5** | **4** | **2** |  | **3** |  |
|  | **TATATTTATATTT** |  |  |  |  |  |  | **1** |  |  |  |  |  |  |  | **1** |  | **1** |  |  |  |  |  |  |  |  |  |  |  |  |  |  |  |  |  | **2** |  | **1** |  |
|  | **TATAAGAA** |  |  |  |  |  |  |  |  |  |  |  |  |  |  |  |  |  |  |  |  |  |  |  |  |  |  |  |  |  |  |  |  |  |  | **1** |  |  |  |
|  | **TATAAAA** |  |  |  |  | **3** |  | **1** |  |  |  |  |  |  |  |  | **1** |  |  |  |  |  |  |  |  |  |  |  | **2** |  |  |  | **2** |  |  |  |  |  |  |
|  | **ccTATAAAaa** |  |  |  |  |  |  |  |  |  |  |  |  |  | **1** |  | **2** |  |  | **1** |  |  |  |  |  |  |  |  |  |  |  | **1** |  |  |  |  |  |  |  |
|  | **TATACA** |  |  |  |  |  |  |  |  |  |  |  |  |  | **1** |  |  | **1** |  | **1** | **1** |  | **1** |  |  |  |  |  |  |  |  |  | **1** | **1** | **2** |  |  |  |  |
|  | **taTATAAAtc** |  |  |  |  |  |  |  |  | **1** |  | **1** |  |  |  |  |  |  |  | **1** |  |  |  |  |  |  | **1** |  |  |  |  |  | **1** |  |  |  |  | **1** |  |
|  | **TATAAATA** |  |  |  |  |  |  |  |  |  |  |  |  |  |  | **1** |  |  |  |  |  |  |  |  |  |  |  |  |  | **1** |  |  | **1** |  |  |  |  | **1** |  |
|  | **taTATAAAg** |  |  |  |  |  |  |  |  | **1** |  |  |  |  |  |  |  |  |  |  |  |  |  |  |  |  |  |  |  |  |  |  |  |  |  |  |  |  |  |
|  | **TATTTAAA** |  |  |  |  |  |  |  |  |  |  |  |  |  |  |  | **1** |  |  |  |  |  |  |  |  |  |  |  |  |  |  |  |  |  |  |  |  |  |  |
|  | **TACATAAA** |  |  |  |  |  |  |  |  |  |  |  |  |  |  |  |  |  |  |  |  |  |  |  |  |  |  |  |  | **1** |  |  |  |  |  |  |  |  |  |
|  | **TATATAA** |  |  |  |  |  |  | **1** |  | **1** |  |  |  |  |  |  |  |  |  | **1** |  |  |  |  |  |  |  |  |  |  |  |  | **3** |  |  |  |  |  |  |
|  | **TAAAGATT** |  |  | **1** |  |  |  |  | **1** | **1** |  |  |  |  | **1** |  |  |  |  |  |  |  |  |  |  |  |  |  |  | **1** |  |  |  |  |  |  |  | **1** |  |
|  | **TACAAAA** | **1** |  |  |  |  |  | **1** |  |  |  |  |  |  |  |  |  |  |  |  |  |  |  |  |  |  |  | **1** |  |  |  |  |  |  |  |  | **1** |  |  |
| **TGA-element** | **AACGAC** |  |  |  |  | **1** | **1** |  |  |  |  |  |  | **1** | **1** | **1** |  |  | **1** |  | **1** |  |  |  |  |  |  |  |  |  |  |  |  |  | **1** |  |  |  | auxin-responsive element |
| **circadian** | **CAAAGATATC** |  |  |  |  |  | **1** |  |  |  |  |  |  |  |  |  | **1** |  |  |  |  |  |  |  | **1** |  |  | **1** |  |  |  |  |  | **1** |  |  | **1** |  |  |
| **ABRE** | **ACGTG** |  | **1** |  | **4** |  |  | **1** | **1** |  |  | **2** | **2** |  | **1** | **1** | **1** |  | **3** | **2** |  |  |  |  | **1** | **1** | **1** | **1** |  | **1** | **2** | **2** |  | **1** | **2** | **2** | **1** | **1** | cis-acting element involved in the abscisic acid responsiveness |
|  | **GACACGTGGC** |  |  |  |  |  |  |  |  |  |  |  |  |  |  |  |  |  | **1** |  |  |  |  |  |  |  |  |  |  | **1** |  |  |  |  |  |  |  |  |  |
|  | **GCAACGTGTC** |  |  |  |  |  |  |  |  |  |  |  |  |  |  |  |  |  |  |  |  |  |  |  |  |  |  |  |  |  |  | **1** |  |  |  |  |  |  |  |
|  | **GCCGCGTGGC** |  |  |  |  |  |  |  |  |  |  |  |  |  |  |  |  |  | **2** |  |  |  |  |  |  |  |  |  |  |  |  |  |  |  |  |  |  |  |  |
|  | **CGCACGTGTC** |  |  |  |  |  |  |  |  |  |  |  |  |  |  |  |  |  | **1** |  |  |  |  |  |  |  |  |  |  |  |  |  |  |  |  |  |  |  |  |
|  | **CACGTG** |  |  |  |  |  |  |  |  |  |  | **2** |  |  | **1** |  |  |  | **2** |  |  |  |  |  |  |  |  |  |  |  |  |  |  | **1** |  |  | **1** |  |  |
|  | **TACGGTC** |  | **1** |  |  |  |  |  |  |  |  |  |  |  |  |  |  |  |  |  |  |  |  |  | **1** |  |  |  |  |  |  |  |  |  |  |  |  |  |  |
|  | **AACCCGG** |  |  |  |  | **1** |  |  |  |  | **1** | **1** |  |  |  |  |  |  |  |  |  |  |  |  |  |  |  |  |  |  |  |  |  |  |  |  |  |  |  |
| **ACE** | **CTAACGTATT** |  | **1** | **1** |  |  |  |  |  |  |  | **1** |  |  | **1** |  |  | **1** | **1** |  |  |  |  |  | **1** |  |  |  |  |  |  |  |  |  |  |  |  |  | cis-acting element involved in light responsiveness |
| **ARE** | **AAACCA** | **2** | **1** |  | **1** | **1** |  | **2** |  | **1** | **4** | **1** | **2** | **1** | **2** | **1** | **1** |  |  | **1** | **1** | **1** |  |  | **3** |  |  | **1** |  |  |  |  |  |  | **1** | **2** |  | **1** |  |
| **Box 4** | **ATTAAT** |  | **1** |  |  | **1** |  | **1** | **3** | **1** |  | **1** |  | **1** |  | **1** |  | **1** |  |  |  | **1** |  |  |  |  |  |  |  | **1** |  |  | **1** | **1** | **1** | **1** |  | **1** |  |
| **CGTCA-motif** | **CGTCA** | **2** | **3** | **3** | **3** |  |  | **3** |  | **3** | **1** | **2** | **1** | **3** |  | **3** |  | **2** | **3** | **2** | **3** |  | **2** | **2** | **2** | **1** | **2** |  |  | **1** | **1** | **2** |  |  | **1** | **4** | **1** | **2** |  |
| **G-box** | **TAACACGTAG** |  |  |  |  |  |  |  |  |  |  | **1** |  |  |  |  |  |  |  |  |  |  |  |  |  |  |  |  |  |  |  |  |  |  |  |  |  |  | cis-acting regulatory element involved in light responsiveness |
|  | **CACGTG** |  |  |  |  |  |  |  | **1** |  |  | **4** |  |  | **2** |  |  |  | **4** |  |  |  |  |  |  |  |  |  |  |  |  |  |  | **2** |  |  | **2** |  |  |
|  | **CACGTC** |  | **1** |  | **5** |  |  | **1** |  |  |  |  | **2** |  |  |  |  |  | **1** | **1** |  |  |  |  |  |  |  |  |  |  |  | **2** |  |  | **3** | **2** |  | **1** |  |
|  | **CACGTT** |  |  |  |  |  |  |  |  |  |  |  |  |  |  | **1** |  |  |  |  |  |  |  |  |  | **1** | **1** | **1** |  | **1** | **1** |  |  |  |  |  |  |  |  |
|  | **CACGAC** |  |  |  |  |  |  |  |  |  | **1** |  |  | **1** |  |  |  |  | **1** | **1** |  |  |  | **1** | **1** |  |  |  |  |  |  |  |  |  |  | **1** |  | **1** |  |
|  | **CAGACGTGGCA** |  |  |  |  |  |  |  |  |  |  |  |  |  |  |  |  |  |  |  |  |  |  |  |  |  |  |  |  |  |  |  |  |  |  |  |  |  |  |
|  | **TAAACGTG** |  |  |  |  |  |  |  |  |  |  |  |  |  |  |  |  |  |  |  |  |  |  |  |  |  |  |  |  | **1** |  |  |  |  |  |  |  |  |  |
|  | **TCCACATGGCA** |  |  |  |  |  |  |  |  |  |  |  |  |  |  |  |  |  |  |  |  |  |  |  |  |  |  |  |  |  | **1** |  |  |  |  |  |  |  |  |
|  | **GCCACGTGGA** |  |  |  |  |  |  |  |  |  |  | **1** |  |  |  |  |  |  |  |  |  |  |  |  |  |  |  |  |  |  |  |  |  |  |  |  |  |  |  |
|  | **CCACGTAA** |  |  |  |  |  |  |  |  |  |  |  |  |  |  |  |  |  |  | **1** |  |  |  |  |  |  |  |  |  |  |  |  |  |  |  |  |  |  |  |
|  | **TACGTG** |  |  |  |  |  |  |  |  |  |  |  |  |  |  |  | **1** |  |  | **1** |  |  |  |  | **1** |  |  |  |  |  | **1** | **1** |  |  |  |  |  |  |  |
|  | **ACACGTGT** |  |  |  |  |  |  |  |  |  |  |  |  |  | **1** |  |  |  | **1** |  |  |  |  |  |  |  |  |  |  |  |  |  |  |  |  |  | **1** |  |  |
| **TC-rich repeats** | **ATTCTCTAAC** |  | **1** |  |  |  |  |  |  |  | **1** |  |  |  |  |  |  | **1** |  |  |  | **1** |  |  |  |  |  |  |  |  |  |  |  |  |  |  |  |  |  |
| **TCA-element** | **CCATCTTTTT** |  | **1** |  |  |  |  |  |  |  |  |  |  |  |  |  |  | **1** |  |  |  |  |  |  |  |  |  |  |  | **1** |  |  | **1** | **1** |  |  |  |  | cis-acting element involved in salicylic acid responsiveness |
|  | **TCAGAAGAGG** |  |  |  |  |  |  |  |  |  |  |  |  | **1** |  |  |  |  |  |  |  |  |  |  | **1** |  |  |  | **1** |  |  |  |  |  |  |  |  |  |  |
| **TGACG-motif** | **TGACG** | **2** | **3** | **3** | **3** |  |  | **3** |  | **3** | **1** | **2** | **1** | **3** |  | **3** |  | **2** | **3** | **2** | **3** |  | **2** | **2** | **2** | **1** | **2** |  |  | **1** | **1** | **2** |  |  | **1** | **4** | **1** | **2** |  |
| **CAT-box** | **GCCACT** | **1** |  | **1** |  | **1** |  |  |  |  |  |  | **1** |  |  | **1** |  |  |  |  | **1** |  | **1** |  |  |  | **2** |  |  |  |  | **2** |  |  | **1** | **1** | **1** |  |  |
| **CCAAT-box** | **CAACGG** |  |  | **1** | **1** |  |  |  |  |  |  |  |  | **2** | **1** |  | **1** |  |  |  | **1** | **1** |  | **2** | **1** |  | **2** |  |  |  |  |  |  |  |  |  |  |  | MYBHv1 binding site |
| **LAMP-element** | **CTTTATCA** |  |  | **1** |  |  |  |  |  |  |  |  |  |  |  | **1** |  |  |  |  |  |  |  |  |  |  |  |  |  |  |  |  |  |  |  |  |  |  | part of a light responsive element |
| **MSA-like** | **TCCAACGGT** |  |  | **1** |  |  |  |  |  |  |  |  |  |  |  |  |  |  |  |  |  |  |  |  |  |  |  |  |  |  |  |  |  |  |  |  |  |  | cis-acting element involved in cell cycle regulation |
| **P-box** | **CCTTTTG** |  |  | **1** |  |  |  |  | **2** |  |  |  |  |  | **1** |  | **1** |  |  |  | **1** |  | **1** |  |  | **1** | **1** | **1** |  |  |  |  |  |  | **1** |  |  |  | gibberellin-responsive element |
| **A-box** | **CCGTCC** |  |  |  | **3** | **1** |  | **1** | **1** |  |  | **1** |  | **1** |  |  |  |  |  |  |  |  |  |  | **1** |  | **2** |  |  |  |  |  | **1** |  |  |  |  | **1** | cis-acting regulatory element |
| **GC-motif** | **CCCCCG** |  |  |  | **1** | **6** |  |  |  |  |  | **1** | **1** |  |  |  |  | **1** | **1** |  |  |  |  | **1** |  | **2** |  | **1** | **1** |  |  | **1** | **1** |  |  | **2** |  |  |  |
| **GTGGC-motif** | **GATTCTGTGGC** |  |  |  | **1** |  |  |  |  |  |  |  |  |  |  |  |  |  |  |  |  | **1** |  |  |  |  | **1** |  |  |  |  |  | **1** |  |  |  |  |  | part of a light responsive element |
| **LTR** | **CCGAAA** |  |  |  | **1** |  |  |  | **2** | **1** | **1** |  |  |  |  |  |  |  | **1** |  |  |  | **1** |  |  |  |  |  |  | **2** |  | **2** |  | **1** |  |  | **1** | **1** |  |
| **Sp1** | **GGGCGG** | **1** |  |  | **1** | **5** |  |  |  | **1** |  | **1** | **2** | **1** |  |  |  |  | **1** |  |  |  |  | **1** |  |  | **5** | **1** |  |  |  | **2** | **1** |  |  | **1** | **1** | **1** | light responsive element |
| **TCCC-motif** | **TCTCCCT** |  |  |  | **1** |  |  |  |  |  |  |  |  |  |  |  | **1** |  |  |  |  |  | **1** |  |  | **1** | **1** |  |  |  |  |  |  |  |  |  | **1** |  | part of a light responsive element |
| **TCT-motif** | **TCTTAC** |  |  |  | **1** |  |  | **1** |  | **1** |  |  | **1** |  |  |  |  |  |  |  |  |  | **1** | **1** |  |  |  | **1** | **1** |  |  |  |  |  |  |  |  | **1** | part of a light responsive element |
| **3-AF1 binding site** | **TAAGAGAGGAA** |  |  |  |  | **1** |  |  |  |  |  |  |  |  |  |  |  |  |  |  |  |  |  |  |  |  |  |  |  |  |  |  |  |  |  |  |  |  | light responsive element |
| **AE-box** | **AGAAACTT** | **2** |  |  |  | **1** |  | **1** |  |  |  |  |  |  |  |  |  |  |  |  |  | **1** |  |  |  |  |  |  |  |  | **1** |  |  |  |  | **1** |  |  | part of a module for light response |
| **GT1-motif** | **GGTTAA** |  |  |  |  | **1** |  |  |  |  |  |  |  |  |  | **1** |  |  |  |  |  |  |  |  |  |  |  |  | **1** | **2** |  |  |  |  |  |  | **1** |  | light responsive element |
| **I-box** | **atGATAAGGTC** |  |  |  |  | **1** |  |  |  |  |  |  |  |  |  |  |  |  |  |  |  |  |  |  |  |  |  |  |  |  |  |  |  |  |  |  |  |  | part of a light responsive element |
|  | **cGATAAGGCG** |  |  |  |  |  |  |  |  |  |  |  |  |  |  |  |  |  |  |  |  |  |  |  | **1** |  |  | **1** |  | **1** |  |  |  |  |  |  |  |  |  |
| **MRE** | **AACCTAA** | **1** |  |  |  |  |  |  | **2** |  |  |  |  |  |  | **1** |  |  |  |  |  |  | **1** |  |  |  |  |  |  | **1** |  |  |  |  |  |  |  |  | MYB binding site involved in light responsiveness |
| **chs-CMA2b** | **GAACCTACACAC** |  |  |  |  |  |  | **2** |  |  |  |  |  |  |  |  |  |  |  |  |  |  |  |  |  |  |  |  |  |  |  |  |  |  |  |  |  |  | part of a light responsive element |
| **GA-motif** | **ATAGATAA** |  |  |  |  |  |  |  | **1** | **1** | **1** |  |  |  |  |  |  |  |  |  |  |  |  |  |  |  |  |  |  |  |  |  |  |  |  |  |  |  | part of a light responsive element |
| **AT-rich sequence** | **TAAAATACT** |  |  |  |  |  |  |  |  |  |  |  |  |  | **1** |  |  |  |  |  |  |  |  |  |  |  |  |  |  |  |  |  |  |  |  |  |  |  |  |
| **AuxRR-core** | **GGTCCAT** |  |  |  |  |  |  |  |  |  |  |  |  |  | **1** |  |  |  |  |  |  |  |  |  |  |  |  |  |  |  |  |  |  |  |  |  |  |  |  |
| **AT1-motif** | **AATTATTTTTTATT** |  |  |  |  |  |  |  |  | **1** | **1** | **1** |  |  |  | **1** |  |  |  |  |  |  |  |  |  |  |  |  |  |  |  |  |  |  |  | **1** |  | **1** | part of a light responsive module |
| **TATC-box** | **TATCCCA** |  |  |  |  |  |  |  |  |  |  |  |  |  |  | **1** |  |  |  |  |  | **1** |  |  | **1** |  |  |  | **1** |  |  |  |  |  |  |  |  |  |  |
| **AT-rich element** | **ATAGAAATCAA** |  |  |  |  |  |  |  |  |  |  |  |  | **1** |  |  | **1** |  |  |  |  |  |  |  |  |  |  |  |  |  |  |  |  |  |  |  |  |  | binding site of AT-rich DNA binding protein (ATBP-1) |
| **ATCT-motif** | **AATCTAATCC** |  |  |  |  |  |  |  |  |  |  |  |  |  |  |  |  |  |  | **1** |  |  |  |  |  |  |  |  |  | **1** |  |  |  |  |  |  |  |  |  |
| **AAAC-motif** | **AATCTAATCC** |  |  |  |  |  |  |  |  |  |  |  |  |  |  |  |  |  |  |  |  |  | **1** |  |  |  |  |  |  |  |  |  |  |  |  |  |  |  | light responsive element |
| **RY-element** | **CATGCATG** |  |  |  |  |  |  |  |  | **1** | **1** |  |  |  |  |  |  |  |  |  |  |  |  |  |  |  |  | **1** |  |  |  |  |  |  |  |  |  | **1** |  |
| **SARE** | **TTCGACCATCTT** |  |  |  |  |  |  |  |  |  |  |  |  |  |  |  |  |  |  |  |  |  |  |  |  |  |  |  |  | **1** |  |  |  |  |  |  |  |  |  |
| **chs-CMA2a** | **TCACTTGA** |  |  |  |  |  |  |  |  |  |  |  |  | **1** |  |  |  |  |  |  |  |  |  |  |  |  |  |  |  |  |  |  |  |  |  |  |  |  | part of a light responsive element |
| **CAG-motif** | **GAAAGGCAGAC** |  |  |  |  |  |  |  |  |  |  |  |  |  |  |  |  |  |  |  |  |  |  |  |  |  |  |  |  |  | **1** |  |  |  |  |  |  |  | part of a light response element |
| **chs-CMA1a** | **TTACTTAA** |  |  |  |  |  |  |  |  |  |  |  |  |  |  |  |  |  |  |  |  |  |  |  |  |  |  |  |  |  | **1** |  |  |  |  |  |  |  | part of a light responsive element |
| **chs-CMA2c** | **ATATACGTGAAGG** | |  |  |  |  |  |  |  |  |  |  |  |  |  |  |  |  |  |  |  |  |  |  |  |  |  |  |  |  | **1** |  |  |  |  |  |  |  | part of a light responsive element |
| **chs-Unit 1 m1** | **ACCTAACCCGG** |  |  |  |  |  |  |  |  |  |  |  |  |  |  |  |  |  |  |  |  |  |  |  |  |  |  |  |  |  |  | **1** |  |  |  |  |  |  | part of a light responsive element |
| **Unnamed__1** | **GAATTTAATTAA** |  |  |  |  |  |  |  |  |  |  |  |  |  |  |  |  |  |  |  |  |  |  |  |  |  |  |  |  |  |  |  | **1** |  |  |  |  |  | 60K protein binding site |
| **Gap-box** | **CAAATGAA(A/G)A** |  |  |  |  |  |  |  |  |  |  |  |  |  |  |  |  |  |  |  |  |  |  |  |  |  |  |  |  |  |  |  |  | **1** |  | **1** |  |  | part of a light responsive element |
| **ATC-motif** | **AGTAATCT** |  |  |  |  |  |  |  |  |  |  | **1** |  |  |  |  |  |  |  |  |  |  |  |  |  |  |  |  |  |  |  |  |  |  |  |  |  |  |  |
| **Box II** | **CCACGTGGC** |  |  |  |  |  |  |  |  |  |  | **1** |  |  |  |  |  |  |  |  |  |  |  |  |  |  |  |  |  |  |  |  |  |  |  |  |  |  | part of a light responsive element |
| **ATCT-motif** | **AATCTAATCC** |  |  |  |  |  |  |  |  |  |  |  |  | **1** |  |  |  |  |  |  |  |  |  |  |  |  |  |  |  |  |  |  |  |  |  |  |  |  |  |
| **MBSI** | **aaaAaaC(G/C)GTTA** | |  |  |  |  |  |  |  | **1** |  |  |  |  |  |  |  |  |  |  |  |  |  |  |  |  |  |  |  |  |  |  |  |  |  |  |  |  |  |

**Table S4 Different features including start and end site of TCS genes in *S. bicolor***

| **Gene Name** | | | | **Start Site** | | | | **End Site** | | | | **Introns** | | | | **Instability index(II)** | | | **Aliphatic index** | | | | **Grand Average of hydropathicity (GRAVY)** | |
| --- | --- | --- | --- | --- | --- | --- | --- | --- | --- | --- | --- | --- | --- | --- | --- | --- | --- | --- | --- | --- | --- | --- | --- | --- |
| **Histidine Kinases (HKs)** |  | | | |  | | | |  | | | |  | | | |  | | | | |  |  |  |
| SbHK1 | | | | 22266130 | | | | 22269754 | | | | 4 | | | | 48.81-unst | | | 91.33 | | | | -0.076 | |
| SbHK2 | | | | 59266327 | | | | 59273161 | | | | 9 | | | | 38.85 | | | 87.58 | | | | -0.179 | |
| SbHK3 | | | | 71489995 | | | | 71495445 | | | | 10 | | | | 38.35 | | | 90.22 | | | | -0.226 | |
| SbHK4 | | | | 8870876 | | | | 8878374 | | | | 10 | | | | 46.05-unst | | | 91.66 | | | | -0.09 | |
| SbHK5 | | | | 55655919 | | | | 55666166 | | | | 13 | | | | 50.78-unst | | | 80 | | | | -0.508 | |
| SbCKl1 | | | | 5136492 | | | | 5152208 | | | | 7 | | | | 42.59-unst | | | 91.31 | | | | -0.055 | |
| SbERS1 | | | | 9801882 | | | | 9806982 | | | | 5 | | | | 37.87 | | | 106.71 | | | | 0.144 | |
| SbETR1 | | | | 5018113 | | | | 5022906 | | | | 5 | | | | 37.48 | | | 106.19 | | | | 0.144 | |
| SbEIN4.1 | | | | 67810845 | | | | 67814687 | | | | 2 | | | | unst-50.1 | | | 100.46 | | | | 0.078 | |
| SbEIN4.2 | | | | 3178416 | | | | 3182964 | | | | 2 | | | | 36.79 | | | 102.41 | | | | 0.088 | |
| SbPHYA | | | | 8713754 | | | | 8721151 | | | | 5 | | | | unst-49.69 | | | 92.6 | | | | -0.151 | |
| SbPHYB | | | | 68035215 | | | | 68043712 | | | | 3 | | | | unst-48.61 | | | 86.72 | | | | -0.167 | |
| SbPHYC | | | | 6748035 | | | | 6753340 | | | | 3 | | | | unst-51.31 | | | 97.24 | | | | -0.112 | |
| **Phosphotransfer Proteins (HPs)** | | |  | | | |  | | | |  | | | |  | | | | |  |  |  |  |  |
| SbHP1 | | | | 19797592 | | | | 19801117 | | | | 5 | | | | 40 | | | 91.52 | | | | -0.079 | |
| SbHP2 | | | | 60641495 | | | | 60644235 | | | | 5 | | | | 26.55 | | | 88.68 | | | | -0.089 | |
| SbHP3 | | | | 62500906 | | | | 62506524 | | | | 6 | | | | 56.36-unst | | | 57.62 | | | | -0.645 | |
| SbHP4 | | | | 55183674 | | | | 55186538 | | | | 5 | | | | 56.83-unst | | | 60.19 | | | | -0.678 | |
| SbHP5 | | | | 8016183 | | | | 8018505 | | | | 4 | | | | 60.23-unst | | | 72.32 | | | | -0.754 | |
| **Response Regulators (RRs)** |  | | | |  | | | |  | | | |  | | | |  | | | | |  |  |  |
| **Type-A RRs** | |  | | | |  | | | |  | | | |  | | | |  | | |  | | |  |
| SbRR4 | | | | 73177403 | | | | 73181260 | | | | 4 | | | | 51.26-unst | | | 83.63 | | | | -0.41 | |
| SbRR9 | | | | 2740967 | | | | 2743306 | | | | 4 | | | | 59.58-unst | | | 79.11 | | | | -0.683 | |
| SbRR10 | | | | 1026753 | | | | 1029205 | | | | 4 | | | | 69.03-unst | | | 83.55 | | | | -0.352 | |
| **Type-B RRs** | |  | | | |  | | | |  | | | |  | | | |  | | |  | | |  |
| SbRR16 | | | | 70434838 | | | | 70438749 | | | | 4 | | | | 45.88-unst | | | 81.3 | | | | -0.424 | |
| SbRR17 | | | | 5416832 | | | | 5421301 | | | | 5 | | | | 39.97 | | | 77.4 | | | | -0.533 | |
| SbRR18 | | | | 66411416 | | | | 66416077 | | | | 5 | | | | 46.1-unst | | | 80.99 | | | | -0.341 | |
| SbRR19 | | | | 72774008 | | | | 72779535 | | | | 5 | | | | 49.94-unst | | | 77.76 | | | | -0.423 | |
| SbRR20 | | | | 5132133 | | | | 5136115 | | | | 5 | | | | 46.69-unst | | | 80.45 | | | | -0.331 | |
| SbRR24.1 | | | | 55120219 | | | | 55130513 | | | | 7 | | | | 43.44-unst | | | 74.46 | | | | -0.459 | |
| SbRR24.2 | | | | 9453701 | | | | 9488726 | | | | 8 | | | | 36.22 | | | 80.69 | | | | -0.536 | |
| **Type-C RRs** | |  | | | |  | | | |  | | | |  | | | |  | | |  | | |  |
| SbRR13 | | | | 3752870 | | | | 3753702 | | | | 1 | | | | 18.5 | | | 96.74 | | | | 0.224 | |
| SbRR14 | | | | 7312249 | | | | 7313602 | | | | 3 | | | | 54.67-unst | | | 88.41 | | | | -0.106 | |
| **Pseudo-RRs** | |  | | | |  | | | |  | | | |  | | | |  | | |  | | |  |
| SbPRR1 | | | | 56625637 | | | | 56628735 | | | | 5 | | | | 52.76-unst | | | 66.37 | | | | -0.623 | |
| SbPRR2.1 | | | | 302694 | | | | 310420 | | | | 5 | | | | 56.94-unst | | | 68.3 | | | | -0.385 | |
| SbPRR2.2 | | | | 8684841 | | | | 8687455 | | | | 5 | | | | 56.48-unst | | | 68 | | | | -0.438 | |
| SbPRR3.1 | | | | 40305107 | | | | 40316802 | | | | 9 | | | | 55.20-unst | | | 70.34 | | | | -0.646 | |
| SbPRR3.2 | | | | 69433326 | | | | 69440358 | | | | 11 | | | | 52.17-unst | | | 59.52 | | | | -0.88 | |
| SbPRR5 | | | | 65784791 | | | | 65790044 | | | | 7 | | | | 52.28-unst | | | 65.3 | | | | -0.827 | |
| SbPRR9 | | | | 4190668 | | | | 4195535 | | | | 7 | | | | 56.89-unst | | | 62.48 | | | | -0.716 | |

| Category | Term | Count | % | PValue | Genes | List Total | Pop Hits | Pop Total | Fold Enrichment | Bonferroni | Benjamini | FDR |
| --- | --- | --- | --- | --- | --- | --- | --- | --- | --- | --- | --- | --- |
| GOTERM_MF_DIRECT | GO:0000155~phosphorelay sensor kinase activity | 10 | 29.41176 | 7.77E-24 | 8155512, 8063415, 8066019, 8060004, 8075742, 8081072, 8069360, 8076011, 8059991, 8086232 | 21 | 12 | 10625 | 421.627 | 6.22E-23 | 6.22E-23 | 6.22E-23 |
| GOTERM_MF_DIRECT | GO:0043424~protein histidine kinase binding | 4 | 11.76471 | 2.28E-08 | 8057993, 8055697, 8075559, 8068945 | 21 | 4 | 10625 | 505.9524 | 1.82E-07 | 6.08E-08 | 6.08E-08 |
| GOTERM_MF_DIRECT | GO:0009927~histidine phosphotransfer kinase activity | 4 | 11.76471 | 2.28E-08 | 8057993, 8055697, 8075559, 8068945 | 21 | 4 | 10625 | 505.9524 | 1.82E-07 | 6.08E-08 | 6.08E-08 |
| GOTERM_MF_DIRECT | GO:0009881~photoreceptor activity | 3 | 8.823529 | 2.02E-05 | 8081072, 8059991, 8086232 | 21 | 4 | 10625 | 379.4643 | 1.61E-04 | 4.03E-05 | 4.03E-05 |
| GOTERM_MF_DIRECT | GO:0003700~transcription factor activity, sequence-specific DNA binding | 6 | 17.64706 | 9.52E-04 | 8058416, 8079408, 8056866, 8072479, 8084640, 8056131 | 21 | 426 | 10625 | 7.12609 | 0.007591 | 0.001523 | 0.001523 |
| GOTERM_MF_DIRECT | GO:0003677~DNA binding | 7 | 20.58824 | 0.001734 | 8058416, 8079408, 8056866, 8072479, 8084640, 8057890, 8056131 | 21 | 730 | 10625 | 4.851598 | 0.013787 | 0.002312 | 0.002312 |
